# Multiscale responses to combined drought and salinity stress in *Apocynum*: a model for climate-smart dryland restoration

**DOI:** 10.3389/fpls.2025.1664532

**Published:** 2026-01-09

**Authors:** Muhammad Rizwan Shoukat, Li Jiang, Zeeshan Ali Buttar, Mohsin Tanveer

**Affiliations:** State Key Laboratory of Desert and Oasis Ecology, Xinjiang Institute of Ecology and Geography, Chinese Academy of Sciences, Urumqi, China

**Keywords:** *Apocynum venetum*, *Apocynum pictum*, abiotic stress, drought-salinity tolerance, dryland restoration, sustainable agriculture, halophytes

## Abstract

Climate change is intensifying the frequency and co-occurrence of abiotic stresses such as drought and salinity, posing serious challenges to crop productivity and ecosystem stability. Traditional research has largely focused on single-stress responses, leaving significant gaps in our understanding of plant resilience under combined and sequential stressors. Species of the genus *Apocynum*, particularly *Apocynum venetum* L. and *Apocynum pictum* Schrenk are naturally adapted to arid and saline environments and offer a valuable model for studying multistress tolerance in non-model species. This review integrates current insights into the morphological, physiological, biochemical, and molecular responses of *Apocynum* under concurrent drought and salinity conditions. Key mechanisms include osmotic adjustment, ion compartmentalization, antioxidant enzyme activation, and stress-induced gene expression involving heat shock transcription factors (HSFs), WRKY transcription factors, NAM, ATAF1/2, and CUC2 transcription factors (NAC), mitogen-activated protein kinase (MAPK) cascades, and flavonoid biosynthesis genes such as *Apocynum venetum* flavanone 3-hydroxylase (*Av*F3H), flavonoid 3′-hydroxylase (*Av*F3’H), and flavonol synthase (*Av*FLS). Additionally, the review highlights the emerging role of stress signaling molecules and phytohormones such as abscisic acid, salicylic acid, and methyl jasmonate in coordinating systemic responses to multiple stressors. Beyond stress resilience, *Apocynum* species provide ecological services including phytoremediation, carbon sequestration, sand dune stabilization, and microbial community restoration. These traits align closely with global restoration goals and support Sustainable Development Goals (SDG) 13 (Climate Action) and 15 (Life on Land). Given their low-input cultivation requirements and multistress tolerance, *Apocynum* species hold promise as climate-smart crops for restoring productivity and resilience in degraded dryland systems. Positioning *Apocynum* as a dual-purpose species that delivers both ecological restoration and crop value can guide the integration of stress-adaptive plants into sustainable agricultural systems under a changing climate.

## 1 Introduction

The accelerating pace of global climate change is intensifying the severity and frequency of abiotic stresses, with drought and salinity ranking among the most destructive constraints to plant productivity (Ali et al., 2025; Raza et al., 2025; Sahu et al., 2025; Wang and Ren, 2025). These stressors increasingly co-occur in arid and semi-arid landscapes, where reduced rainfall, elevated evapotranspiration, and rising soil degradation result in synergistic impacts on plant performance (Alsubih et al., 2025; Iqbal and Yaning, 2024; Ji et al., 2025; Tariq et al., 2024). The combined stress of drought and salinity exerts compounding physiological and biochemical disruptions inducing osmotic imbalance, ion toxicity, oxidative damage, and metabolic disarray that are more harmful than either stress alone (Alptekin and Kunkowska, 2025; Sithole et al., 2025). Despite significant advances in abiotic stress biology, current research remains heavily skewed toward isolated stress scenarios (Jiang et al., 2025c). Consequently, there is a knowledge gap in how plants coordinate defense under dual stress, particularly across levels of organization from whole-plant to molecular levels, including signaling crosstalk and resource allocation (Alptekin and Kunkowska, 2025). Moreover, widely used model species such as *Arabidopsis thaliana* (L.) Heynh. and *Oryza sativa* L. lack the intrinsic stress tolerance needed for survival in extreme habitats, limiting their translational relevance to real-world applications in degraded ecosystems (Saini et al., 2025; Yaschenko et al., 2025). Thus, identifying naturally resilient, non-model plants with multiscale stress tolerance under arid conditions is critical for developing sustainable agricultural and ecological restoration strategies (Salse et al., 2024). *Apocynum* L. is a small genus in the dogbane family Apocynaceae, placed in subfamily Apocynoideae and tribe Apocyneae (Endress et al., 2014; Livshultz et al., 2018). Depending on the authority, about four to five species are currently accepted, with a named hybrid in North America; Eurasian taxa include *Apocynum venetum* L. and *Apocynum pictum* Schrenk (syn. *Apocynum hendersonii* Hook.f.), and North American taxa include *Apocynum androsaemifolium* L. and *Apocynum cannabinum* L (Johnson et al., 1998; Xiang et al., 2023).

The genus has a broadly Holarctic native range across temperate Asia, southeastern Europe, and North America, with *Apocynum venetum* especially widespread in temperate Asia and parts of Europe (Ditommaso et al., 2009; Xiang et al., 2023). Morphologically, *Apocynum* are perennial, rhizomatous herbs with milky latex; opposite leaves; cymose inflorescences; small campanulate to urceolate corollas; and paired, slender follicles bearing silky-tufted seeds (Ditommaso et al., 2009; Fishbein et al., 2018; Xiang et al., 2023). Among such candidates, *Apocynum* species including *Apocynum venetum* (*Av*), *Apocynum pictum* (*Ap*), and *Apocynum cannabinum* (*Ac*) stand out as perennial halophytes adapted to drought and salt prone environments (Gao et al., 2019a, 2019; Jiang et al., 2021a, 2025; Shao et al., 2022; Zhao et al., 2025). *Av* (Luobuma in Chinese), in particular, has gained attention for its ecological plasticity, bast fiber suitable for textiles and composites, standardized flavonoid extracts, and biochar or energy recovery from processing residues, medicinal uses, and low-input growth profile in degraded soils across Central Asia and northwestern China (Jiang et al., 2021a, 2021; Kim et al., 2025; Xie et al., 2025; Xiu et al., 2025). These plants have evolved a sophisticated array of morphological, physiological, biochemical, and molecular mechanisms that collectively confer resilience under combined abiotic stress (Li et al., 2023c; Xiang et al., 2023; Zhou and Li, 2023). Work on other halophytes such as *Salicornia* L., *Atriplex* L., and *Suaeda* Forssk. ex J.F.Gmel. documents conserved salinity strategies, including Na^+^ exclusion through salt overly sensitive (SOS) and high-affinity K^+^ transporter 1 (HKT1) modules and salt excretion or storage by salt glands and bladders, offering useful comparators for *Apocynum* (Chen et al., 2024; Muhammad et al., 2024; Zhou et al., 2024). Morphologically, *Apocynum* species exhibit xeromorphic features such as thick sclerophyllous leaves, waxy cuticles, and sunken stomata, which minimize water loss while maximizing water-use efficiency. Deep fibrous root systems enhance drought avoidance and soil stabilization in arid or saline substrates (Jiang et al., 2021b). Physiologically, these plants sustain photosynthesis and gas exchange under stress through stomatal regulation and osmotic adjustment, achieved via the accumulation of compatible solutes like proline and soluble sugars (Li et al., 2023a, 2023; Wang et al., 2019; Xu et al., 2021). Biochemically, they activate enzymatic antioxidants, including superoxide dismutase (SOD), peroxidase (POD), catalase (CAT), and ascorbate peroxidase (APX), and non-enzymatic antioxidant defenses including flavonoids like hyperoside, quercitrin, and kaempferol which protect against oxidative stress and modulate signaling cascades (Jiang et al., 2021b; Xiang et al., 2023; Xie et al., 2025; Xiu et al., 2025). Under salinity, *Av* upregulates stress-associated myeloblastosis transcription factors (MYB), WRKY, and NAC transcription factors with SOS-pathway involvement, modulates ApHKT1 to limit Na^+^ influx and stabilize the K^+^/Na^+^ ratio, and induces flavonoid-biosynthesis genes such as *Av*F3H, *Av*F3′H, and *Av*FLS that improve tolerance in heterologous tests (Xu et al., 2021; Zhang et al., 2023; Zhao et al., 2025; Zhen et al., 2024). Under drought, PEG-based and tissue-level assays in *Av* show coordinated induction of MYB/WRKY/NAC families and osmotic adjustment via proline and soluble sugars, with antioxidant enzymes and flavonoids acting together; AvNAC genes also show stress- and tissue-specific expression under both drought and salt (Huang et al., 2023; Wu et al., 2022). These traits synergistically preserve cellular homeostasis and ensure survival under dual stress (Jiang et al., 2025b; Wang et al., 2019; Wu et al., 2022; Xu et al., 2021; Zhao et al., 2025).

Beyond its physiological adaptations, *Apocynum* species provides critical ecosystem services that support land restoration and climate resilience (Ping et al., 2014; Thevs et al., 2012). Previous studies show that *Av* plantations improve soil structure, reduce wind erosion, and enhance microbial biomass outcomes vital for reversing desertification and restoring ecological function in drylands (Günther et al., 2017; Jiang et al., 2021b; Ping et al., 2014; Rouzi et al., 2017; Thevs et al., 2012). Additionally, *Apocynum* exhibits phytoremediation capacity by sequestering excess sodium (Na^+^) and trace metals such as lithium (Li^+^) in vacuoles, helping reclaim saline or contaminated soils (Jiang et al., 2019, 2018). Its deep root systems enhance carbon sequestration, stabilize soils, and minimize runoff, supporting nature-based solutions for soil health and mitigation of climate extremes. Cultivation across degraded regions like Xinjiang, China, highlights its value in large-scale reforestation (Geng et al., 2025; Jiang et al., 2021b). Collectively, these findings indicate that *Apocynum* is a low-input restoration candidate with measurable contributions to SDG 13 and SDG 15 (Mccarthy et al., 2025). Economically, *Apocynum* supports climate-smart bioeconomy pathways. Its bast fibers offer exceptional tensile strength, suitable for technical textiles and composites, while its flavonoid-rich leaves, especially hyperoside and isoquercitrin are used in functional teas and herbal medicines (Xie et al., 2025; Xiu et al., 2025). Additionally, *Apocynum*-based honey exhibits antimicrobial activity, enhancing its nutraceutical value (Jiang et al., 2024). These applications promote circular bioeconomy integration, allowing full-plant utilization for fiber, pharmaceuticals, and biomass valorization (Jiang et al., 2021b; Shao et al., 2022; Xiang et al., 2023). Crucially, its cultivation on saline soils avoids food crop competition while supporting rural livelihoods in resource-limited environments (Nair et al., 2025).

Despite these multifaceted benefits, research on *Apocynum* remains fragmented. Most studies have examined isolated traits fiber tensile strength, phytochemical composition, or abiotic stress physiology without integrating these into a comprehensive framework for multistress tolerance under real-world conditions. Moreover, the use of omics-based approaches (transcriptomics, metabolomics) to dissect gene regulatory networks in *Apocynum* is still emerging. There remains a critical need to consolidate and contextualize existing findings across disciplines, linking stress adaptation mechanisms to applied strategies for dryland rehabilitation and sustainable agriculture. Therefore, this review aims to: (1) synthesize the multiscale adaptive mechanisms morphological, physiological, biochemical, and molecular of *Apocynum* under combined drought and salinity stress; and (2) evaluate its ecological and bioeconomic roles in climate-smart land restoration, including soil stabilization, phytoremediation, carbon sequestration, and circular bioeconomy pathways. By narrowing the focus to these two core objectives, the review offers a cohesive foundation for deploying *Apocynum* as a model species in resilient agriculture and sustainable land management and their alignment with Sustainable Development Goals (SDGs 13 and 15).

## 2 *Apocynum* species as multifunctional assets for sustainable arid land development

**Figure 1:**
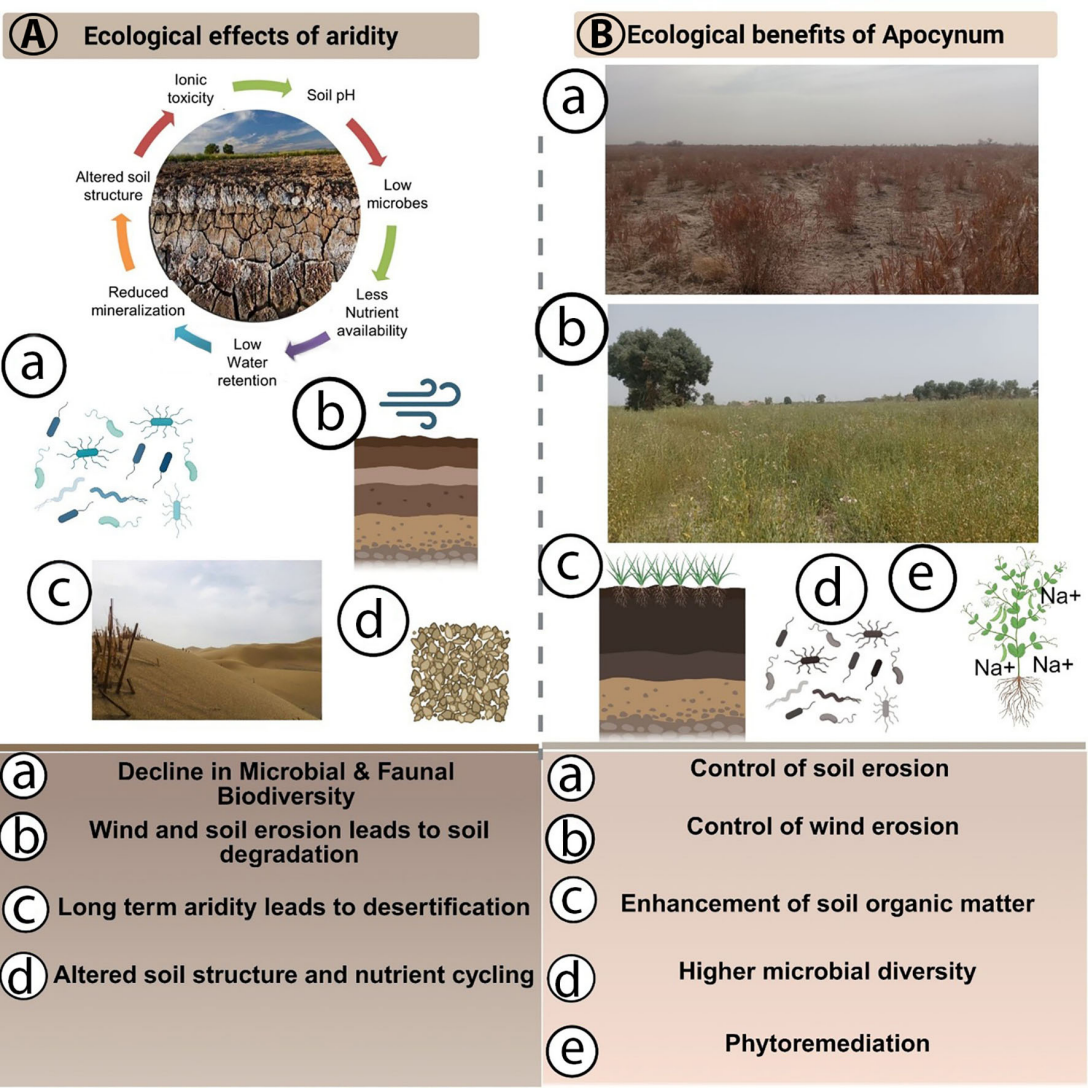
Ecological effects of aridity and ecological benefits of *Apocynum* in degraded arid lands. **(A)** Aridity-driven degradation of soil and biota, including reduced water retention, ionic toxicity, lower microbial abundance, altered soil structure and nutrient cycling, and progression toward desertification, summarized as **(a)** decline in microbial and faunal biodiversity, **(b)** wind and soil erosion leading to soil degradation, **(c)** long-term aridity leading to desertification and **(d)** altered soil structure and nutrient cycling. **(B)** Ecological benefits of *Apocynum* stands in arid fields: **(a,b)** contrast between degraded bare land and vegetated *Apocynum* field, **(c)** improved soil cover and structure, **(d)** higher microbial diversity and **(e)** phytoremediation through uptake and sequestration of excess salts. Together, these panels illustrate how *Apocynum* cultivation can mitigate key symptoms of arid land degradation by stabilizing soil, enhancing soil quality and supporting soil biota.

*Apocynum* species, especially *Av*, offer dual benefits for arid economies through ecological restoration and economic utilization. **Table 1** synthesizes species-level information on native range, habitat, stress tolerance, bast-fiber quality and leaf flavonoid content for the main *Apocynum* taxa, highlighting the broad ecological plasticity and multi-purpose value of *Av* compared with *Ap* and *Ac*. **Figure 1** builds on this comparison by schematically linking these traits to ecosystem services (soil stabilization, phytoremediation, carbon sequestration) and value chains (technical fiber, functional teas, biochar and honey), illustrating how *Apocynum* cultivation can transform saline, nutrient-poor land into climate-smart production systems that support SDGs 2, 8, 13 and 15 (Jiang et al., 2021a, 2021; Su et al., 2025; Xiang et al., 2023).

**Table 1:** Ecological and economic traits of major *Apocynum* species relevant to arid and saline-land use.

Species name	Key features & uses	distribution/Adaptation	Notable compounds/Bioactivity	References
*Apocynum venetum*	Medicinal (antihypertensive, antioxidant, antidepressant), fiber crop, tea plant	Arid, saline soils in China, Central Asia	High flavonoid & phenolic content, hyperoside, isoquercitrin, strong antioxidant activity	(Kim et al., 2025; Xiang et al., 2023; Xie et al., 2025, 2012; Zhao et al., 2025)
*Apocynum pictum*	Fiber crop, medicinal, ecological restoration, drought/salt tolerant	Tarim Basin, Central Asia, Northswest China	Stress-responsive genes, flavonoids, used for saline land restoration	(Jiang et al., 2021a, 2021; Thevs et al., 2012; Xie et al., 2024a; Zheng et al., 2022)
*Apocynum cannabinum*	Traditional medicinal uses, source of apocynin (acetophenone)	North America	Apocynin (NADPH oxidase inhibitor), anatomical xeromorphism	(Bhatia et al., 2025; Savla et al., 2021)

The table compares native range, typical habitats, stress-tolerance characteristics, bast-fiber properties and leaf flavonoid profiles for *Apocynum venetum*, *Apocynum pictum*, *Apocynum cannabinum* and related taxa. These contrasts highlight the broad ecological plasticity and multi‑functional value of *Av* for combined restoration and bioeconomy applications.

### 2.1 Comparative ecological advantage of *Apocynum* over other halophytes

Compared to other salt-tolerant plants or halophytes such as *Salicornia* L. and *Atriplex* L., *Av* is better suited for long-term ecosystem functions, offering deep soil stabilization, bioresource production, and higher phytochemical content (Jiang et al., 2021a; Manzoor et al., 2022; Xie et al., 2012). **Table 2** makes this comparison explicit by juxtaposing *Av* with other halophyte species in terms of (i) tolerated salinity ranges and water requirements, (ii) documented effects on erosion control, soil organic carbon and microbial biomass, and (iii) main harvested products and market uses. In arid landscapes, *Apocynum* reduces wind erosion and sand drift through its deep (2–3 m) fibrous root systems and dense canopy, making it effective for sand fixation. Field trials in Xinjiang showed wind erosion was reduced by 70–89% in planted plots. When interplanted with *Caragana korshinskii* Kom., it enhanced soil microbial respiration and promoted rhizosphere symbioses, aiding soil health recovery (Liu et al., 2022; Ping et al., 2014; Sun and Chen, 2025). In desertified zones, soil organic carbon (SOC) increased by 1.2 to 2.8 times compared to bare sand dunes (Li et al., 2022). Its deep roots and litter inputs promote microbial processes such as carbonate precipitation and organic matter stabilization, contributing to soil fertility and structure (Gao et al., 2018b; Li et al., 2021; Wang et al., 2024). In arid landscapes, field studies show that *Av* plantings substantially reduce erosion, enhance carbon storage, and improve soil condition. In the plain desert of the Altay Region in Xinjiang, planting *Av* markedly enhanced both carbon storage and erosion control. Plant carbon density increased by about 340% and 460% compared with the plain desert, leading to roughly 21 to 23% higher total ecosystem carbon density. At the same time, wind erosion modulus decreased by about 71 to 89% and soil water erosion by around 14 to 18% in *Av* plots relative to bare control plots, while surface stability and vegetation cover improved markedly (Ping et al., 2014). Along saline basins and riparian zones in northwestern China, stands of *Av* and *Ap* established on degraded or saline soils are reported to have higher soil organic matter and richer soil biota than adjacent uncultivated or conventionally cropped land, highlighting clear soil amelioration benefits in addition to fiber and leaf production (Jiang et al., 2021a, 2021; Thevs et al., 2012; Xiang et al., 2023). At the plant level, experiments under saline–alkali stress show a roughly 40% increase in the root cortex to diameter ratio, about a 100% enlargement of xylem vessel diameter, nearly 70% thickening of phloem fiber walls, and around 50% increases in root soluble sugars and root flavonoid content in *Av* changes that support more efficient water uptake, salt exclusion, and mechanical stability in degraded soils (Zhang et al., 2025b).

**Table 2:** Comparative performance of *Apocynum venetum* and representative halophytes in arid systems.

Halophytes	Ecological benefits	Economic value	Stress tolerance	References
*Apocynum venetum*	Stabilizes soil, enhances biodiversity, and improves soil quality	Medicinal, fiber, honey production.	High drought and salinity tolerance.	(Jiang et al., 2021a; Manzoor et al., 2022; Xie et al., 2012)
*Aeluropus littoralis*	Contributes to soil stabilization and habitat for wildlife	Potential use in biofuel production and as forage.	High salt tolerance and ability to recover post-stress.	(Hashemipetroudi et al., 2022)
*Thellungiella halophila*	Supports biodiversity in saline environments	Investigated for its potential in saline agriculture.	Superior salt tolerance compared to glycophytes; can thrive in extreme salinity.	(Pang et al., 2010)
*Halogeton glomeratus*	Enhances soil structure and prevents erosion	Used in livestock grazing and as a potential bioenergy source.	Tolerates high salinity through ion compartmentalization.	(Wang et al., 2015)
*Nitraria tangutorum*	Plays a role in desert reclamation and soil improvement	Harvested for its edible fruits and medicinal properties.	Exhibits mechanisms for both salt and drought tolerance.	(Pan et al., 2022)
*Salicornia europaea*	Important for coastal ecosystem health and habitat provision	Cultivated for its edible shoots and potential in biofuel production.	High tolerance to salinity and ability to adapt to varying salt concentrations.	(Hrynkiewicz et al., 2019)
*Atriplex halimus*	Enhances soil fertility and provides habitat for various species	Used for forage, soil reclamation, and as a windbreak.	Exhibits cross-tolerance to salinity and drought.	(Nemat Alla et al., 2011)

Key metrics include tolerated salinity ranges, water‑use characteristics, reported impacts on erosion control, soil organic carbon and microbial biomass, and primary harvested products. The comparison illustrates why *Apocynum venetum* is a strong candidate for multi‑functional restoration where both ecosystem services and marketable biomass are required.

These *Apocynum*-specific findings are consistent with broader evidence from arid and semi-arid afforestation. A global synthesis of tree and shrub planting on degraded land reports mean increases of about 131% in soil organic carbon stocks and 88% in total nitrogen when barren land is converted to woody vegetation, underlining the potential of perennial halophytes such as *Apocynum* to act as soil-carbon sinks while restoring fertility (Liu et al., 2018). From a climate and resource-use perspective, life-cycle assessment indicates that cotton fiber in China has a climate footprint of about 4.43 kg CO_2_-equivalents per kilogram and a phosphorus footprint near 101 g P kg^−1^, whereas *Apocynum* fiber can be produced at roughly 1.93 kg CO_2_-equivalents and 1.6 g P kg^−1^, suggesting large potential savings in greenhouse gas emissions and nutrient pollution if *Apocynum* partially substitutes cotton in Xinjiang (Günther et al., 2017). Farm-level economic analysis along the Tarim Basin further shows that *Ap* cultivation requires about 16,250 yuan ha^−1^ in costs, generates around 49,000 yuan ha^−1^ in revenue, and yields profits near 32,800 yuan ha^−1^, outperforming cotton and Chinese red date under comparable conditions (Rouzi et al., 2017). Taken together, these quantitative data demonstrate that *Apocynum*-based systems can simultaneously deliver windbreak and sand-fixation services, enhance soil carbon and nutrient stocks, and provide economically competitive fiber and leaf production in arid, saline landscapes. The next section, comparative genomics and traits of *Apocynum* species, compares the genomic and physiological capacities that underlie these field outcomes.

### 2.2 Comparative genomics and traits of *Apocynum* species

*Av* and *Ap* are the best studied in the genus, with evidence for phytoremediation, medicinal use, and bast fiber quality (Jiang et al., 2021a; Xiang et al., 2023). Chloroplast genome analyses reveal high conservation between *Av* and *Ap*, both having plastid genomes of ~150,800 bp in size and sharing the same number of protein-coding genes (86), though *Ap* contains more SSRs, indicating greater organelle-level variability (Guo et al., 2022; Zheng et al., 2022). Available nuclear assemblies report ~23,147 predicted protein-coding genes in *Ap* versus ~20,292 in *Av*, with more than 91% of models functionally annotated; counts may vary with assembly completeness and annotation pipelines (Xie et al., 2024b). Comparative stress-response genomics show that *Av* upregulates flavonoid biosynthesis genes such as *AvFLS* and *AvF3H*, while *Ap* relies on a broader network of WRKY transcription factors (Xie et al., 2024b; Zhang et al., 2023). Building on the plastome and nuclear patterns already noted, independent datasets suggest the two species rely on different levers when salinity coincides with osmotic limitation. In *Av*, salt stress elevates phenylpropanoid and flavonoid enzyme activity, including *Av*F3H and *Av*FLS, which strengthens antioxidant capacity and helps protect membranes (Li et al., 2023a; Zhang et al., 2023). By contrast, the *Ap* genome reports an expanded set of regulators and broad induction of WRKY transcription factors under water-related stress, consistent with faster reprogramming of ion transport and osmolyte balance (Xie et al., 2024a). Physiologically, *Av* demonstrates higher photosynthetic performance (12–18 μmol CO_2_ m^-2^ s^-1^) and superior water-use efficiency compared to traditional crops like cotton (Cui et al., 2019; Li et al., 2023a). Both *Av* and *A. pictum* show notable phytoremediation capacity, including lithium uptake from contaminated soils (Jiang et al., 2018; Xiang et al., 2023). Bast fibers from *Av* have higher tensile strength (450–600 MPa), and its leaves contain elevated levels of bioactive compounds like quercitrin and hyperoside, contributing to superior antioxidant potential (Chan et al., 2015; Gao et al., 2019a; Shao et al., 2022). These biochemical traits correlate with differential expressions of phenylpropanoid pathway genes (Chen et al., 2023, 2020; Dorjee et al., 2024). These patterns translate into ecosystem effects only when stress is active in the field, so the next subsection evaluates contributions to climate mitigation and soil remediation.

### 2.3 *Apocynum* in climate mitigation and soil remediation

**Figure 2:**
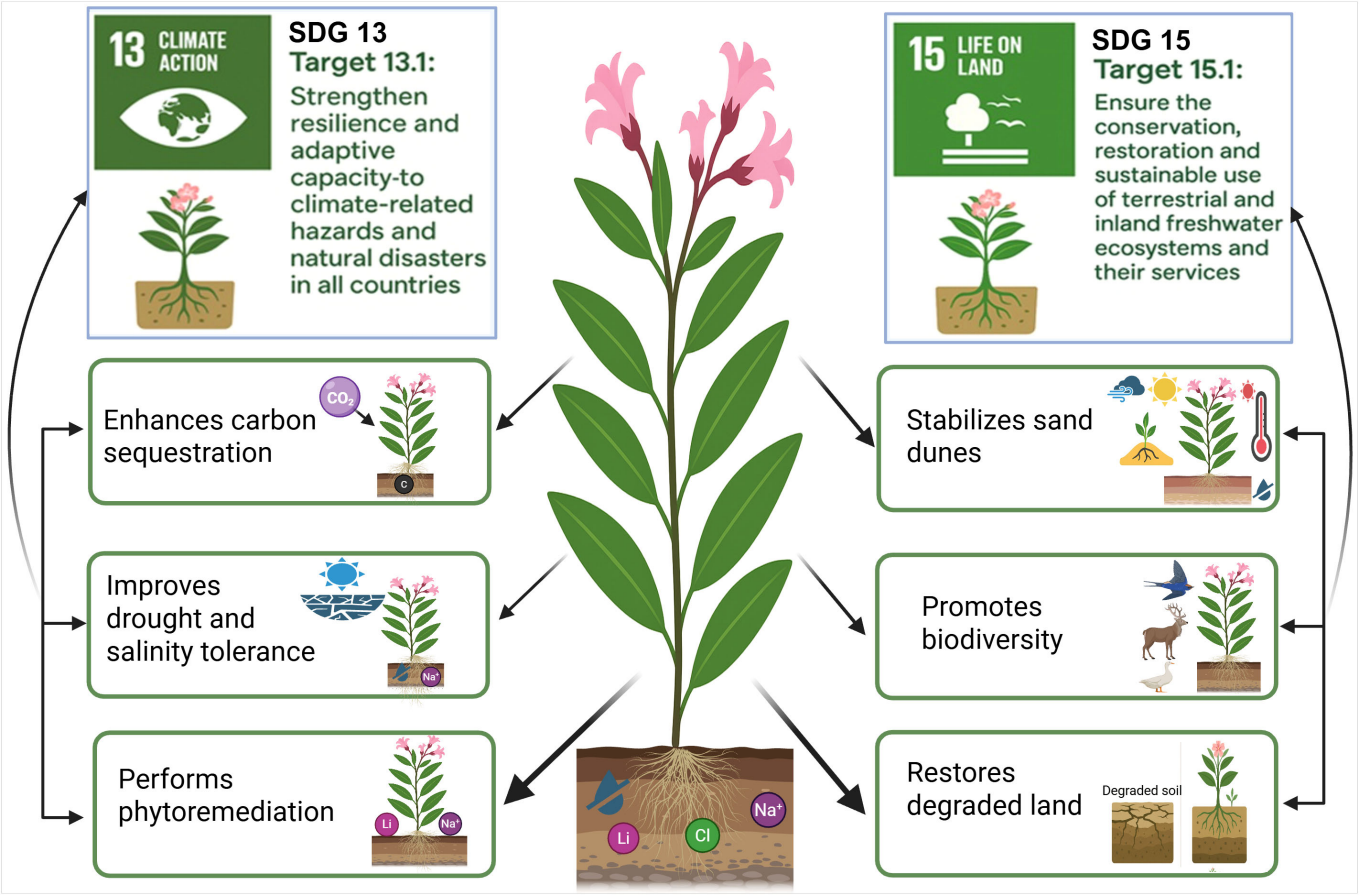
Ecosystem services provided by *Apocynum* stands in arid saline landscapes and their links to Sustainable Development Goals 13 and 15. The central *Apocynum* plant with deep roots represents belowground storage and ion sequestration, while surrounding panels illustrate key functions: enhancing carbon sequestration, improving drought and salinity tolerance, performing phytoremediation through uptake of excess Na^+^, Li^+^ and Cl ions, stabilizing sand dunes, promoting aboveground biodiversity and restoring degraded land. Together, these services contribute to climate resilience (SDG 13, Climate Action) and land restoration and conservation (SDG 15, Life on Land) in dryland regions.

Halophyte plantings on degraded soils commonly can reduce wind erosion, improve soil organic matter, and support richer microbial communities (Hu et al., 2024; Li and Liu, 2020; Wang et al., 2021). Increased evapotranspiration from halophyte vegetation can also contribute to local cooling in arid environments (Freedman et al., 2014). Where persistent cover is achieved, *Apocynum* may show comparable outcomes and thus support United Nations SDG 13 (Climate Action) alongside land-restoration goals. **Figure 2** synthesizes these pathways for *Apocynum* by diagramming how deep rooting, litter inputs and rhizosphere processes drive carbon sequestration and soil restoration, while ion uptake and vacuolar sequestration underpin phytoremediation of Na^+^ and Li^+^. The figure also summarizes how these biophysical functions may translate into biodiversity support and climate-risk reduction at the landscape scale. *Av* deep root systems help secure water during dry periods and play a role in enhancing soil stability and preventing erosion in arid lands (Jiang et al., 2021a; Wu et al., 2022; Xiang et al., 2023), supporting SDG 2 (Zero Hunger) by improving soil productivity. Beyond physical stabilization, *Av* and *Ap* exhibit phytoremediation potential. These species accumulate toxic ions like Na^+^ and Li^+^ without compromising growth, transporting them to vacuoles via membrane transporters. Root uptake and leaf compartmentalization have been documented with bioconcentration factors (BCF) and translocation factors (TF) >1, confirming their value for salt and lithium-contaminated soil remediation (Jiang et al., 2019, 2018, 2021; Kot, 2018; Li et al., 2023a; Qiao et al., 2018), further advancing SDG 15 (Life on Land). Given these regulating services, it is important to assess market and public-health value, so the next subsection summarizes economic and health benefits.

### 2.4 Economic and health benefits of *Apocynum*

Economically, *Apocynum* bast fibers contain 65–75% cellulose and low lignin, yielding high mechanical performance, with tensile strength up to 401.56 cN dtex^−1^, surpassing cotton and flax (Jiang et al., 2021b; Lou et al., 2020). Its fibers are used in eco-textiles and composites (Chen et al., 2022; Xu et al., 2020a). Because *Apocynum* can be cultivated on non-arable land, it offers income opportunities without competing with food crops (Thevs et al., 2012; Xiang et al., 2023). Additionally, specialty honey derived from *Apocynum* nectar shows broad-spectrum antimicrobial and antioxidant activity, with more than 85% inhibition against E. coli and *S. aureus* (Arunasree et al., 2024; Xiang et al., 2023), supporting applications in wound care, functional foods, and therapeutic products (Al-Kafaween et al., 2023; Dahiya et al., 2024). Flavonoid-rich tea and extracts, containing rutin and quercetin, are reported to provide neuroprotective, hepatoprotective, and cardioprotective benefits (Jiang et al., 2019; Xie et al., 2024b; Zeng et al., 2022). Taken together, these outcomes support SDGs 1, 2, 3, 9, and 12. Recognizing *Apocynum’s* ecological and economic significance, the following section details its specific contributions to key SDGs, particularly in the context of land restoration and climate action. To connect products with sustainability outcomes, the final subsection outlines circular bioeconomy strategies that reduce waste and raise resource efficiency.

### 2.5 Circular bioeconomy strategies for achieving SDGs

In addition to their ecological and agronomic contributions, *Apocynum* species, especially *Av* can contribute to SDG 12 (Responsible Consumption and Production) when circular practices are applied, for example full-plant valorization of stems for fiber, leaves for standardized extracts, and residues for energy or biochar, with water reuse where feasible. The entire plant can be utilized to generate high-value products while enhancing carbon sequestration and minimizing waste (Jiang et al., 2021a, 2021; Rouzi et al., 2017; Thevs et al., 2012; Xiang et al., 2023). For instance, cellulose-rich stems can be converted into textiles or nanocellulose composites, and flavonoid-rich leaves into pharmaceutical extracts (Dorjee et al., 2024; Shao et al., 2022). Residual biomass can undergo anaerobic digestion or pyrolysis to produce biochar, which at 10–20 t/ha can sequester carbon for centuries and improve soil water retention by 15–20% (Ali and Al-Wahaibi, 2025; Jiang et al., 2025a; Kabir et al., 2023; Khan et al., 2024; Kocsis et al., 2022; Ndede et al., 2022). Deep root systems complement this by contributing to belowground carbon sinks (Buss et al., 2021; Kell, 2011). Wastewater from fiber extraction, which is rich in residual polyphenols, can be treated with biochar filtration to recover polyphenols by adsorption onto the biochar followed by solvent or mild alkaline desorption, enabling water reuse and sorbent regeneration (Abid et al., 2022; Dahani et al., 2025; El Barkaoui et al., 2025; Olugbenga et al., 2024; Wang et al., 2022). Such circular processes enhance the role of *Apocynum*-based systems as nature-based solutions, integrating environmental sustainability with resource efficiency. The resulting closed-loop system maximizes resource efficiency and reduces environmental impact core priorities under SDG 12 (Cho et al., 2024; Olugbenga et al., 2024). Applied studies on *Salicornia* highlight product safety and processing considerations for edible shoots, which are relevant to *Apocynum* supply-chain design (Marangi et al., 2024).

## 3 Arid survival: *Apocynum* stress adaptive responses

This section explains the mechanistic basis of *Apocynum* tolerance to aridity by organizing evidence across morphology, physiology, and gene regulation, quantifying typical response ranges, and noting which findings have or have not been validated outside controlled conditions.

### 3.1 Salinity tolerance mechanisms

*Av* demonstrates multifaceted tolerance to salinity through a coordinated interplay of ion transport regulation, antioxidant defense, osmotic adjustment, and gene expression reprogramming. *Apocynum* responses to salinity have been quantified under controlled assays, which helps interpret the signaling steps involved. At the seed stage, NaCl treatments from 0 to 300 mmol L^−1^ produced clear, dose-dependent shifts: activities of SOD, POD, and CAT increased up to about 150 mmol L^−1^ and then declined toward 300 mmol L^−1^, while APX increased across the entire range; soluble sugar, malondialdehyde, and proline contents increased with rising NaCl, and protein content decreased at higher concentrations, all measured after short-term germination assays (Li et al., 2023c). Seedling and hydroponic studies report consistent patterns at 100 to 350 mmol L^−1^ NaCl, including elevated root SOD and CAT and transcriptome enrichment for MAPK, phytohormone signaling, ion transport, and cell-wall processes. Typical assay ranges are 0, 100, 200, and up to 350 mmol L^−1^ NaCl with ~7-day exposure after a short ramp (Li et al., 2023c; Xu et al., 2021). Salt also induces the flavonoid pathway, with increased expression of AvF3H and AvFLS and higher flavonol accumulation linked to improved salt tolerance in heterologous tests (Zhang et al., 2023). A critical component of this adaptation is the maintenance of Na^+^/K^+^ homeostasis, which mitigates ion toxicity and preserves cellular function. Under saline conditions, Na^+^ competes with K^+^ for binding sites and enzymatic cofactors, impairing protein function, membrane potential, and photosynthesis (Cheeseman, 2013; Zhao et al., 2020). While Na^+^ can serve a limited osmotic function, excessive accumulation is toxic and must be tightly regulated. Studies in *Apocynum* reveal that Na^+^ content significantly increases in roots, stems, and leaves under prolonged salt stress. However, salt-tolerant species like *Ap* maintain a higher K^+^/Na^+^ ratio under control conditions, though this advantage declines with increasing salinity (Gao et al., 2023a; Xu et al., 2021). Interestingly, tetraploidy in *Av* does not significantly alter ion distribution patterns, contrasting with species like *Zoysia japonica* Steud. or *Lycopersicon pennellii* (Correll) D’Arcy, suggesting a distinct regulatory mechanism in *Apocynum* (Li et al., 2023d; Tahal et al., 2000). Key insights from hydroponic experiments indicate that cultivation method and stress duration significantly influence ion profiles *Apocynum* grown under uniform, long-term salt exposure displayed consistent Na^+^ accumulation trends, reflecting field-relevant stress physiology (Xu et al., 2021). Moreover, *Apocynum* appears to prefer vacuolar sequestration of Na^+^ mediated by tonoplast-localized transporters like *Av* Na^+^/H^+^ antiporter 1 (AvNHX1*)* as a strategy to reduce cytosolic toxicity while maintaining osmotic balance (Bai et al., 2023; Qin et al., 2024; Waheed et al., 2024). Across halophytes, coordination between HKT1 transporters and SOS signaling helps maintain K^+^ to Na^+^ homeostasis, consistent with ion-balance responses reported for *Apocynum* (Hualpa-Ramirez et al., 2024; Zhou et al., 2024). Across osmotic and saline conditions, *Av* maintains effective K^+^ uptake; added NaCl raises leaf Na^+^ for osmotic adjustment while K^+^ remains the main contributor, supporting photosynthesis and growth (Cui et al., 2019). In model plants, root HKT1 limits Na^+^ movement to shoots and NHX1 sequesters Na^+^ in vacuoles, helping sustain lower Na^+^:K^+^ ratios (Abdelaziz et al., 2017; Almeida et al., 2017; Liu et al., 2025; Wang et al., 2019). Consistent with this response in *Av*, NAC genes such as *Av*NAC58 and *Av*NAC69 show strong tissue-specific induction near 200 mM NaCl (Huang et al., 2023).

A crucial molecular regulator of Na^+^ and K^+^ dynamics is the high-affinity potassium transporter gene *ApHKT1*. Functional studies revealed that *ApHKT1* expression is highest in roots under mild salinity (50 mM NaCl) and is lower at 100–200 mmol L^−1^ in seedling or hydroponic time courses (Assaha et al., 2017; Gao et al., 2023a; Xu et al., 2021), which strongly correlates with salt tolerance across *Apocynum* species. Salt-tolerant *Ap*, for example, displays higher root expression of *Ap*HKT1 under mild stress, facilitating controlled Na^+^ exclusion and K^+^ retention, while sensitive species exhibit reduced expression and greater Na^+^ accumulation in leaves, correlating with higher toxicity (Assaha et al., 2017; Xu et al., 2021). With increased salt concentrations (100–200 mM NaCl), *Ap*HKT1 expression is progressively suppressed particularly in roots indicating an adaptive mechanism to limit Na^+^ influx during severe stress. The spatial expression gradient of *ApHKT1* higher in roots and lower in leaves/phloem helps limit Na^+^ transport to aerial tissues, protecting photosynthetically active cells from ion overload (Gao et al., 2023a). Supporting this are coordinated upregulations of other ion-related genes, including *TPK1*, *HAK*, and *SOS2* (Salt Overly Sensitive 2), which enhance K^+^ uptake and Na^+^ exclusion, stabilizing ion ratios under stress (Zhao et al., 2025). The role of SOS signaling is particularly relevant here: it operates in parallel with *Ap*HKT1 to prevent Na^+^ cytotoxicity and preserve membrane potential (Wang et al., 2019; Xu et al., 2021; Zhao et al., 2025). These findings align with mechanisms described in *Arabidopsis thaliana*, *Triticum aestivum*, and *Oryza sativa*, but show species-specific modulation in *Apocynum* (Gholizadeh et al., 2024). In conjunction with ion regulation, *Av* mobilizes antioxidant defenses under salinity, increasing activities of enzymes such as SOD, CAT, and POD, and accumulating osmoprotectants like proline and soluble sugars. Furthermore, flavonoid biosynthesis is upregulated through genes such as *AvFLS* and *AvF3H*, which enhance quercetin and kaempferol levels molecules known for their dual function as antioxidants and signaling regulators (Wang et al., 2021; Zhang et al., 2023). Overexpression of *AvFLS* in tobacco increased total flavonoids and improved salt-stress performance, while heterologous expression of *AvF3H*, *AvF3′H*, and *AvFLS* in Arabidopsis elevated total flavonoids and rutin and enhanced salt-tolerance readouts (Wang et al., 2021; Zhang et al., 2023). Altogether, salinity tolerance in *Av* arises from a network of root-localized Na^+^ transport suppression (via ApHKT1), vacuolar sequestration (*AvNHX1*), K^+^ homeostasis (TPK1, HAK), ROS scavenging (SOD, flavonoids), and osmotic balance (proline, sugars), all underpinned by hormone signaling and transcription factor activation (Zhao et al., 2025). This multilayered mechanism positions *Av* as a promising model for breeding or engineering salt-tolerant crops for marginal lands.

### 3.2 Drought tolerance mechanisms

*Av* demonstrates a remarkable capacity to tolerate drought through a combination of morphological plasticity, physiological regulation, and stress-responsive gene networks that together maintain cellular homeostasis and water-use efficiency. Morphologically, the species develops deep and extensive root systems capable of penetrating lower soil layers to access subsurface moisture reserves, a trait critical for sustaining water uptake during extended dry periods (Li et al., 2023a; Thevs et al., 2012; Xiang et al., 2023). Concurrently, its leaves exhibit xeromorphic features such as thickened cuticles, dense wax layers, and sunken stomata that minimize transpirational water loss. These sclerophyllous leaf traits, often accompanied by heliotropic reorientation, help reduce solar exposure and thermal stress under arid conditions (Abubakar et al., 2022; Cui et al., 2019; Jiang et al., 2021a). At the physiological level, *Av* maintains photosynthetic activity during water scarcity through rapid and reversible stomatal closure, which limits transpiration while protecting the integrity of the photosynthetic apparatus (Pradhan et al., 2025; Wu et al., 2022). The plant performs osmotic adjustment by accumulating compatible solutes such as proline and soluble sugars, which function to stabilize osmotic potential, preserve membrane structure, and protect cellular proteins (Parvin et al., 2025; Zhao and Dai, 2015). These osmolytes also serve as metabolic reservoirs during recovery from dehydration. Under seedling drought assays, SOD, POD, and CAT activities rise relative to controls, with APX increases reported where assayed in *Av* (Iqbal et al., 2023; Li et al., 2023c; Wu et al., 2022). Similar antioxidant trends under water deficit are also noted by Iqbal et al. (2023). Together, osmotic regulation and ROS scavenging enhance cellular resilience during desiccation. Comparative halophyte studies show that drought and salt interact to shape gas exchange, ionic balance, and antioxidant capacity, aligning with the *Apocynum* responses (Muhammad et al., 2024; Zandalinas and Mittler, 2022).

Beyond metabolic adjustments, *Apocynum* modulates gas exchange and water use under drought. In seedlings exposed to PEG near −0.2 MPa, supplying 25 mM NaCl kept leaf K as the main contributor to osmotic potential, improved relative water content, and supported higher photosynthetic activity, consistent with tighter control of stomatal conductance under water deficit (Cui et al., 2019). Water-use efficiency in this review refers to A_n_/g_s_, where A_n_ is the leaf net CO_2_ assimilation rate and g_s_ is stomatal conductance to water vapor; units are µmol CO_2_ m^−2^ s^−1^ for A_n_ and mol H_2_O m^−2^ s^−1^ for g_s_, so A_n_/g_s_ has units µmol CO_2_ per mol H_2_O (Busch et al., 2024; Seibt et al., 2008). This framing highlights the role of stomatal kinetics: faster opening raises carbon gain when light increases, whereas rapid closure conserves water during declines or transient stress (Bertolino et al., 2019; Lawson and Vialet-Chabrand, 2019). At the whole-plant scale, root phenotypes that combine lower root hydraulic conductance with longer axes and dense root hairs can delay soil limitation and sustain transpiration in drying soils, providing a useful lens for interpreting *Apocynum* drought performance (Cai et al., 2022).

Hormonal signaling, particularly via abscisic acid (ABA), plays a central role in coordinating drought responses in *Av*. ABA not only promotes stomatal closure to conserve water but also induces the formation of deeper root systems that improve access to soil water (Cui et al., 2019; Xu et al., 2018). This dual function enhances both short-term drought avoidance and long-term drought tolerance. Additionally, the plant exhibits elevated accumulation of stress-related flavonoids (e.g., hyperoside, quercitrin), which act as non-enzymatic antioxidants and modulators of stress signaling pathways (Chan et al., 2015; Shao et al., 2022; Xu et al., 2020b). These specialized metabolites increase during drought, especially in leaf tissues, contributing to both ROS detoxification and membrane stabilization. Molecular analyses further reveal the complexity of *Av* drought response. Transcriptome profiling under PEG-induced drought stress during germination has identified differentially expressed genes involved in hormone signaling (ABA, gibberellins, auxin, ethylene), cellular detoxification, and transcriptional regulation (e.g., WRKY, NAC, MYB) (Wu et al., 2022; Zhang et al., 2025a). Key upregulated genes such as GASA4, PP2CA, and EIN3 mediate hormonal crosstalk that fine-tunes root development, leaf growth suppression, and stomatal behavior. This coordinated gene regulation supports physiological resilience during early developmental stages and enables successful seedling establishment under drought conditions. Importantly, *Av* also reallocates resources under drought stress, shifting biomass toward lignified stems and deeper roots while reducing leaf expansion and shoot elongation. This redistribution enhances mechanical support, reduces water demand, and prioritizes resource acquisition and storage in roots traits particularly valuable for perennial species in arid regions (Jiang et al., 2021a; Li et al., 2023a). Taken together, these interconnected drought tolerance mechanisms ranging from structural traits to metabolite synthesis and gene regulation demonstrate how *Av* thrives in marginal ecosystems and reinforces its suitability for ecological restoration and low-input cultivation in arid lands.

### 3.3 Combined drought and salinity tolerance mechanisms

**Figure 3:**
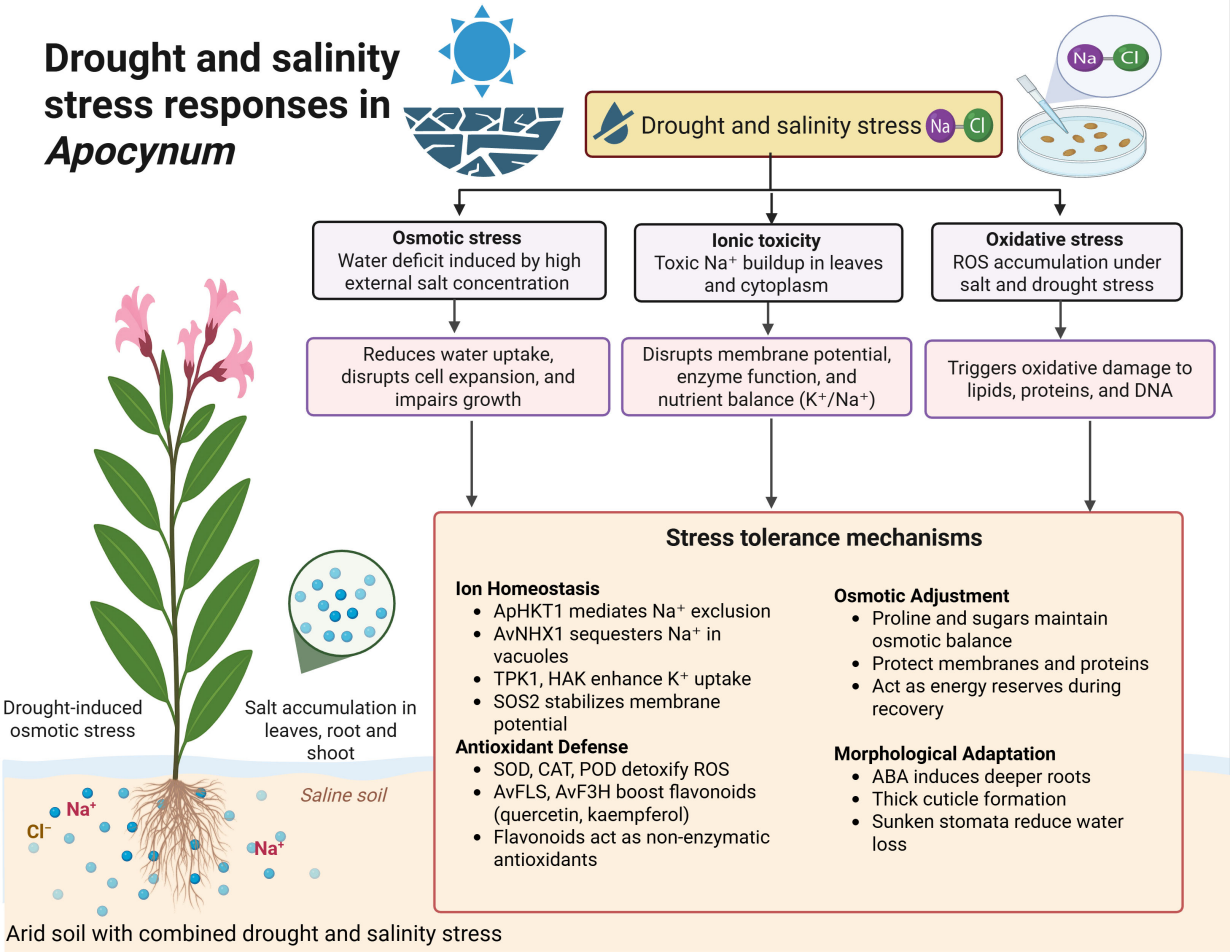
Integrated morphological, physiological and biochemical responses of *Av* to combined drought and salinity stress. The schematic first shows how water deficit and high external NaCl induce osmotic stress, ionic toxicity and oxidative stress, which reduce water uptake, disrupt membrane potential and trigger ROS damage. The lower panel groups major tolerance mechanisms into four modules: ion homeostasis (ApHKT1 mediated Na^+^ exclusion, AvNHX1 vacuolar sequestration, TPK1 and HAK enhanced K^+^ uptake, SOS2 stabilized membrane potential), antioxidant defense (SOD, CAT, POD and flavonoid biosynthesis enzymes such as AvFLS and AvF3H), osmotic adjustment (proline and soluble sugars) and morphological adaptation (deep roots, thick cuticle, sunken stomata). Together, these cross protective mechanisms help *Av* survive under dual drought and salinity stress.

Recent omics syntheses indicate that combined stresses rewire networks rather than simply add single-stress effects, which helps interpret *Apocynum* responses under concurrent drought and salinity (Khan et al., 2025; Secomandi et al., 2025; Zandalinas and Mittler, 2022). In arid and semi-arid regions, plants are frequently exposed to simultaneous drought and salinity stress, which together impose osmotic imbalance, ion toxicity, and oxidative stress. *Av* has evolved a highly integrated and dynamic defense system that enables it to cope with such combined abiotic stress. These stressors act synergistically to disrupt water uptake, impair Na^+^/K^+^ balance, and induce the generation of reactive oxygen species (ROS), collectively threatening cellular integrity and metabolic function (Cheeseman, 2013; Zhao et al., 2020, 2025). **Figure 3** provides an integrated overview of these responses in *Av*, linking root and shoot traits, osmotic adjustment, antioxidant activity and ion homeostasis to whole-plant performance under dual stress. Building on the individual tolerance mechanisms described in the previous sections, the figure emphasizes cross-protective strategies such as coordinated ABA signaling, vacuolar Na^+^ sequestration and flavonoid accumulation that underpin survival and persistence in marginal ecosystems. The morphological foundation of *Av* stress resilience namely, deep and fibrous root systems, sclerophyllous leaves, thick cuticles, and sunken stomata remains essential under combined stress (Jing et al., 2024). These traits, previously highlighted under both drought and salinity responses, are further reinforced by adaptive heliotropic leaf reorientation and biomass allocation favoring lignified stems and root tissues. Such structural plasticity enhances subterranean water extraction while reducing irradiance exposure and evaporative water loss (Cui et al., 2019; Jiang et al., 2021a; Li et al., 2023c; Wu et al., 2022).

Physiologically, *Av* exhibits intensified osmotic adjustment through elevated proline and soluble sugar accumulation, which stabilizes cell turgor and protects cellular macromolecules under combined osmotic and ionic stress. These osmolytes also support continued metabolic function during dehydration. Concurrently, the enzymatic antioxidant machinery comprising SOD, POD, CAT, and APX works in concert with non-enzymatic antioxidants (e.g., flavonoids) to detoxify ROS generated by dual stress exposure (Li et al., 2023c; Wu et al., 2022). As previously detailed, these mechanisms are central to *Av* oxidative stress tolerance under individual stress conditions, but under combined drought and salinity they are co-activated at higher magnitude and with longer duration. Hormonal pathways, especially ABA, continue to serve as the principal signal integrators during combined stress. ABA promotes rapid stomatal closure, enhances root elongation, and activates downstream transcription factors and stress-inducible genes. In addition to ABA, jasmonic acid, ethylene, and auxin pathways further modulate cell wall remodeling, antioxidant gene expression, and growth suppression under combined stress, amplifying the fine-tuned crosstalk described in the drought section (Cui et al., 2019; Xu et al., 2018; Zhang et al., 2023).

#### 3.3.1 Signaling crosstalk under combined drought–salinity stress

**Figure 4:**
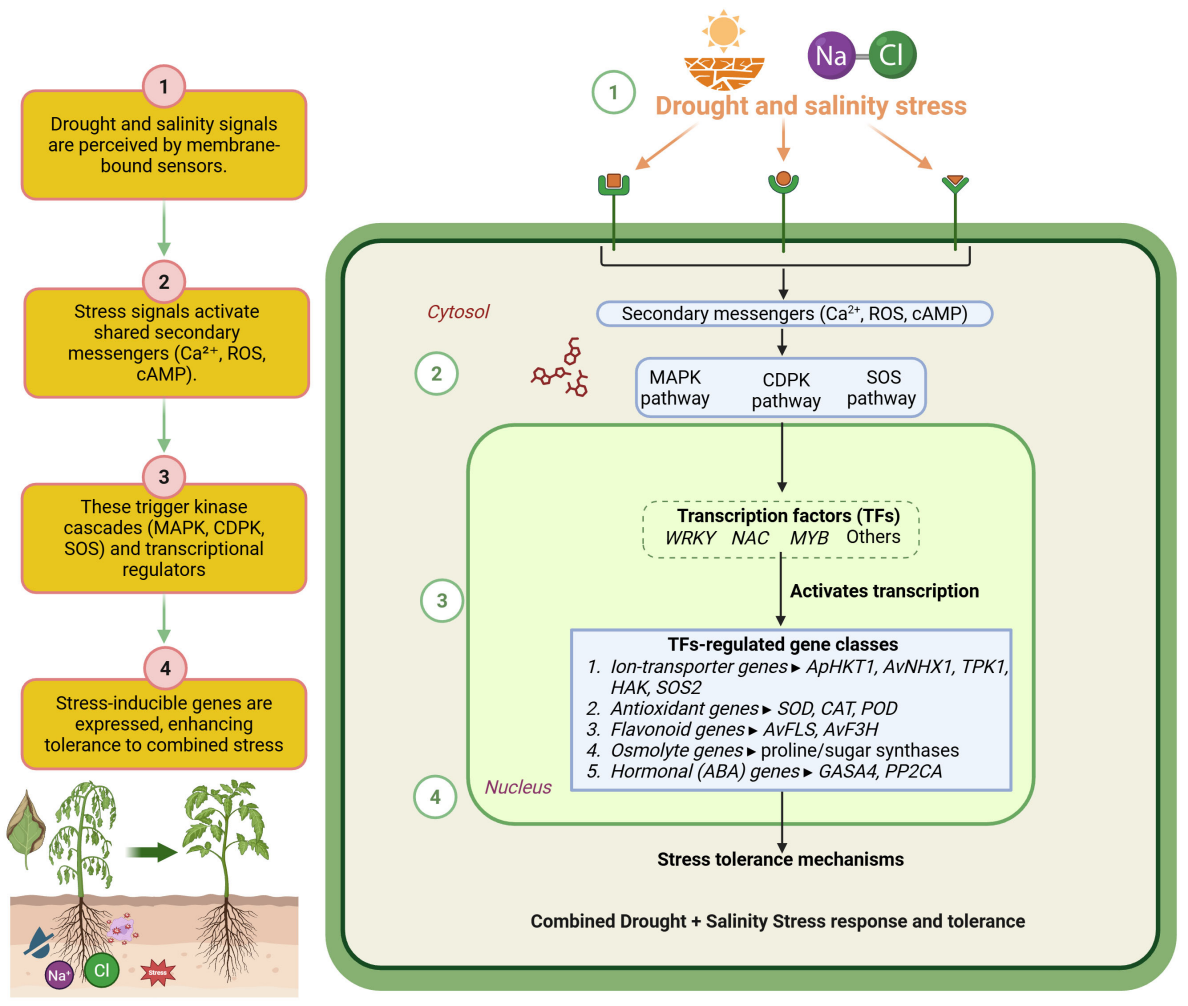
Conceptual signaling network underlying *Apocynum* responses to combined drought and salinity stress. On the left, a four-step overview shows how (1) drought and salinity signals are perceived by membrane-bound sensors, (2) shared secondary messengers (Ca²^+^, ROS, cAMP) are activated, (3) these messengers trigger kinase cascades (MAPK, CDPK, SOS) and transcriptional regulators, and (4) stress-inducible genes are expressed, enhancing tolerance to combined stress. Within the cell (right panel), secondary messengers converge on transcription factor families (WRKY, NAC, MYB, and others) that activate ion-transporter genes (ApHKT1, AvNHX1, TPK1, HAK, SOS2), antioxidant genes (SOD, CAT, POD), flavonoid biosynthesis genes (AvFLS, AvF3H), osmolyte genes (proline and sugar synthases) and ABA-related regulators (GASA4, PP2CA). Coordinated regulation of these gene modules maintains Na^+^/K^+^ homeostasis, osmotic balance, ROS detoxification, and growth under combined drought and salinity stress.

Work in Arabidopsis and crops shows that combined drought–salinity does not simply add single stress responses but reconfigures signaling networks in non-additive ways, with both synergistic and antagonistic regulation of downstream targets (Zandalinas and Mittler, 2022). Under such multifactorial stress, ABA accumulation, Ca²^+^ spikes and ROS waves converge on protein kinase cascades (MAPKs, CDPKs) and the Salt Overly Sensitive (SOS) module, which together modulate HKT and NHX type transporters to adjust Na^+^/K^+^ balance and stomatal behavior; at the same time, some growth-promoting hormone outputs are attenuated, reflecting trade-offs between survival and growth (Yang and Guo, 2018; Zandalinas and Mittler, 2022). Current data indicate that Apocynum venetum recruits similar modules but with halophyte-specific tuning. Salt-stress transcriptomes and WGCNA identify NAC, WRKY and MYB transcription factors, SOS-related components and ion transport genes as core regulators of the response (Li et al., 2023c; Zhen et al., 2024). Element–metabolite profiling of *Av* and *Ap* seeds further shows that elevated K^+^, Ca²^+^, and antioxidant metabolites, including flavonoids, underpin superior salt tolerance (Jiang et al., 2025b). As summarized in **Figure 4**, this tolerance mechanism relies on the specific intersection of ABA signaling and the SOS pathway. Signals perceived by membrane sensors activate shared secondary messengers (Ca²^+^, ROS, cAMP) which reconfigure downstream kinase cascades to drive the expression of ion transporters (ApHKT1, AvNHX1, SOS2), antioxidant enzymes, and flavonoid biosynthesis genes (*AvFLS*, *AvF3H*). This synergistic strengthening of Na^+^ exclusion, vacuolar sequestration, and ROS scavenging, while down-weighting growth pathways distinguishes the combined stress state from single drought or single salinity.

Ion homeostasis is a pivotal challenge under combined salinity and drought stress due to compounding ionic and osmotic disturbances. *Av* addresses this by integrating its previously described strategies: suppression of *ApHKT1* expression in roots to limit Na^+^ influx under high salinity, and vacuolar sequestration of Na^+^ through *AvNHX1* to reduce cytosolic toxicity. This process is complemented by upregulation of TPK1, HAK, and SOS2, which help preserve K^+^ uptake and facilitate Na^+^ exclusion (Bai et al., 2023; Waheed et al., 2024; Xu et al., 2021). The spatial expression pattern of *ApHKT1* higher in roots and lower in leaves/phloem supports a tissue-specific strategy that minimizes Na^+^ accumulation in photosynthetic tissues (Gao et al., 2023b). While these mechanisms are individually functional under saline conditions, under combined stress, their regulation becomes more dynamic and coordinated to respond to fluctuating external osmotic conditions. At the molecular level, combined stress triggers complex signaling networks involving Ca²^+^, ROS, and kinase cascades (MAPK and CDPK), which activate key transcription factors such as WRKY, NAC, and MYB. Under combined drought and salinity, signaling through Ca²^+^, ROS, and kinase cascades (MAPK and CDPK) integrates with SOS-mediated ion homeostasis to drive gene expression by transcription factors controlling osmolyte biosynthesis, ROS detoxification, and ion transport. **Figure 4** details the proposed signaling network, showing how Ca²^+^, ROS, and MAPK/CDPK cascades converge on WRKY, NAC and MYB transcription factors and downstream ion transporters (ApHKT1, AvNHX1, TPK1, HAK, SOS2) and flavonoid/osmolyte biosynthesis genes, thereby coordinating Na^+^/K^+^ homeostasis and oxidative-stress defense under multifactorial stress. Flavonoid biosynthesis genes identified under salt stress are co-induced during combined stress, leading to enhanced accumulation of antioxidant flavonoids like quercetin and kaempferol (Wang et al., 2021; Zhang et al., 2023). These compounds serve dual roles in ROS scavenging and stress signaling.

Collectively, these integrated morphological, physiological, biochemical and molecular adaptations enable *Av* to maintain cellular function, growth, and metabolic integrity under dual stress conditions. The convergence of stress signaling, ion regulation, and antioxidant defense across organ systems provides a model of multi-layered resilience. Moreover, these responses are not isolated events but are systemically coordinated across tissues and time, allowing *Av* to dynamically balance growth with survival in fluctuating arid and saline environments. These characteristics position *Av* as a valuable species for ecological restoration and as a functional genomic model for developing climate-resilient crops in saline and drought-prone landscape.

## 4 Cultivation and propagation of *Apocynum* species in arid and saline soils

Arid lands typically have low water-holding capacity, high salinity, and frequent drought, making conventional cultivation difficult (Colunga et al., 2025). However, *Apocynum* has shown adaptability through techniques like drip irrigation, nutrient application, and optimized propagation methods (Gao et al., 2023b, 2018; Jiang et al., 2021b). This section highlights practical methods to enhance its production in arid zones.

### 4.1 Agronomic basis and site-specific cultivation conditions

The viability of *Apocynum*, particularly *Av*, in harsh, saline, and low-fertility soils is well established (Jiang et al., 2021b; Li et al., 2023b; Xiang et al., 2023). Native to Eurasia and North America, it thrives in degraded, saline-alkaline, and sandy soils (Jiang et al., 2021a, 2021b). Field trials in Xinjiang confirm its minimal irrigation requirements under <300 mm rainfall (Gao et al., 2018a, 2018; Jiang et al., 2021a, 2021; Ma et al., 2024; Xiang et al., 2023; Zhang et al., 2022a, 2022). *Av* and *Ap* tolerate salinity and grow well where conventional crops fail (Gao et al., 2023b). Seeds germinate optimally at 25–30°C (Rong et al., 2015), and can establish under drought or salinity (Jiang et al., 2021a). Unlike cotton, *Av* survives on groundwater (Thevs et al., 2012). Supplemental irrigation boosts yields in coarse soils (Gao et al., 2023b). Drip irrigation every 7 days improves water-use efficiency (Gao et al., 2023b). PEG-induced drought simulations enhanced seedling performance (Wu et al., 2022). While suited to nutrient-poor soils, balanced fertilization (34.8 kg/ha N, 57.2 kg/ha P) boosts biomass and flavonoids (Li et al., 2023b). Both *Av* and *Ap* show phytoremediation capacity (Chen et al., 2020; Jiang et al., 2021a; Li et al., 2023a, 2023, 2023), with dual outputs of fibers and bioactive compounds (Prates‐Valério et al., 2019). Compared to jute, hemp, and cotton, *Apocynum* offers greater resilience, lower inputs, and dual-purpose utility (Chen et al., 2022; Xiang et al., 2023; Xie et al., 2024b). Its cultivation is suitable for saline regions and climate-adapted systems (Cui et al., 2019; Günther et al., 2017). Its ecological advantages and economic returns make it a model crop for sustainable bioeconomies (Jiang et al., 2021b; Li et al., 2023b; Rouzi et al., 2017; Wang et al., 2007). These foundational insights into site-specific cultivation guide the selection of practical propagation techniques suited for stress-prone environments. For propagation planning, Kanuma soil is optimal for cutting propagation, while saline and saline-alkali soils are highly suitable for field establishment and seed or rhizome propagation of *Apocynum* species (Jiang et al., 2018, 2021; Seo et al., 2023). *Av* is supported for high fiber and leaf-flavonoid production on sandy to sandy-loam saline or saline-alkaline soils, with successful field management under drip irrigation (Gao et al., 2018a, 2018) and improved leaf yield and quality under balanced fertilization (Gao et al., 2019a, 2019; Li et al., 2023b; Xiang et al., 2023). *Ap* fits sites where salinity fluctuates or where lithium occurs, with demonstrated Li accumulation and growth on alkaline, low-organic-matter desert topsoil (Jiang et al., 2018). *Ap* is a strong option for higher-salinity or saline-alkali flats and saline-soil improvement, supported by comparative genome-metabolome evidence for stress-tolerance modules relevant to restoration (Jiang et al., 2021b; Li et al., 2023a).

### 4.2 Cultivation techniques for *Apocynum* species

Propagation techniques include direct sowing, rhizome propagation, cuttings, and transplanting. Direct sowing is cost-effective and beneficial for desertification control but is limited by environmental stress (Jiang et al., 2021a, 2021). Adverse rainfall may hinder germination (Gavric and Omerbegovic, 2021). Though transplanting has better seedling establishment, it’s more labor-intensive (Caporale et al., 2022; Turebayeva et al., 2022). Rhizome propagation supports stress resilience (Jiang et al., 2021a) but requires careful handling (Gao et al., 2019b). Cutting propagation, especially in *A. lancifolium*, performs well with Kanuma soil and IBA treatments (Seo et al., 2023; Zhou et al., 2021), supporting mass propagation (Turebayeva et al., 2022). Transplanting nursery-raised seedlings with optimized substrate and NPK pre-treatment enhances establishment (Kyalo et al., 2020; Oyedeji et al., 2014; Reis et al., 2025). These complementary techniques enable scalable and sustainable cultivation under arid stress. A clear understanding of the agricultural cycle and harvesting windows is also essential to maximize both ecological services and economic returns. **Table 3** collates this practical information by contrasting seed, rhizome, cutting and transplant-based propagation systems, the associated substrate and irrigation requirements, and the recommended harvest stages for fiber and leaf biomass. The table is intended as a decision-support tool for selecting site-appropriate cultivation strategies in saline and drought-prone environments.

**Table 3:** Overview of cultivation and propagation options for *Apocynum* species in arid and saline soils.

Aspect	Ideal condition/Key features	Influencing factors/notes	Notable insights	References
Seed storage	Use freshly matured or 1-year-old seeds for best germination in saline soils	Seed viability, storage duration, salinity tolerance	Short-term storage enhances germination and stress resilience	(Jiang et al., 2021a)
Germination	10/25°C or 15/30°C (Av/*Ap*); 34°C (*Ac*)	Seed age, temperature, moisture	Fresh seeds under moderate stress offer best results	(Jiang et al., 2021a; Li et al., 2023c; Webster and Cardina, 1999; Wu et al., 2022)
Vegetative Growth	Spring–Summer; perennial/semi-shrub	Salinity, drought, propagation method	Strong adaptive traits for saline and arid zones	(Cahill Jr et al., 2002; Jiang et al., 2021b; Tan et al., 2024)
Flavonoid Accumulation	Peaks in August (field); day 25 (lab)	Growth stage, environment	Peak flavonoid content aligns with ideal harvest window	(Shao et al., 2022; Zhang et al., 2022a)
Reproduction/Harvest	Late summer–early fall; August for highest flavonoid content	Harvest timing based on plant use and compound content	Harvest timing optimizes bioactive yield and fiber quality	(Shao et al., 2022)
Soil	Saline, poor, or degraded soils	Soil salinity, organic matter content	Adapted to grow in poor, saline soils where other crops fail	(Jiang et al., 2021a, 2018, 2021)
Water	Moderate moisture; drip irrigation preferred	Irrigation method, water availability	Efficient water uses in low-precipitation environments	(Jiang et al., 2021a; Li et al., 2023b, 2023)
Fertilizer	N2P2: 34.8 kg/ha N + 57.2 kg/ha P	Nutrient type and application rate	Enhanced productivity with targeted nutrient application	(Li et al., 2023b)
Propagation	Fresh seeds or cuttings in Kanuma soil + Rootone	Seed freshness, rooting hormones, substrate type	Effective propagation supports establishment in stress-prone areas	(Jiang et al., 2021a; Seo et al., 2023)
Direct Sowing	Low cost, scalable, improves soil health	Low germination in harsh sites, weed control challenges	Ideal for low-input dryland rehabilitation	(Farooq et al., 2011; Jiang et al., 2021a, 2021)
Transplanting	Higher seedling establishment and early growth	Labor-intensive, costly, requires careful seedbed management	Suitable for smallholder intensification	(Biswas, 2020; Lampayan et al., 2015; Navarro García et al., 2011; Sánchez-Blanco et al., 2004)
Cutting Propagation	High genetic fidelity, uniform and rapid propagation	Limited to species with good rooting; sensitive to environment	Useful for elite genotype multiplication	(Bowman and Albrecht, 2017; Druege, 2020; Weingarten et al., 2024)

This summary includes germination requirements, soil and water preferences, optimal flavonoid harvesting windows, fertilizer regimes, and propagation strategies (direct sowing, transplanting, and cutting propagation) relevant to establishing *Apocynum* under arid and saline conditions.

The table summarizes seed-storage recommendations, germination conditions, rhizome and cutting protocols, nursery versus direct-sowing approaches, irrigation regimes and optimal harvest windows for fiber and leaf biomass. These management guidelines provide a practical basis for scaling *Apocynum*-based systems in degraded landscapes.

### 4.3 Agricultural cycle and harvesting

Field and system-level work in other halophytes offers practical guides for saline agriculture, from Salicornia aquaculture integration to *Atriplex* forage and phytoremediation trials (Giménez et al., 2024; Kachout et al., 2025). *Apocynum* species regenerate annually from rhizomes. Sowing starts in mid-spring, with seed germination optimized under 10/25°C to 15/30°C (Jiang et al., 2021a; Li et al., 2023b; Wu et al., 2022). Shoot emergence occurs by March–April (Abubakar et al., 2022). Their tolerance to stress is linked to redox balance and flavonoid accumulation (Jiang et al., 2021b; Tan et al., 2024). Full establishment may take two years, after which regular harvesting begins (Jiang et al., 2021b). The perennial life cycle includes flowering (June–August) and fruiting (September–October). Flavonoids peak in August, making it ideal for medicinal harvest (Jiang et al., 2021b; Shao et al., 2022; Xiang et al., 2023). Fiber is harvested in late summer or winter, reaching peak yield in the third year and maintaining productivity for up to a decade (Abubakar et al., 2022; Rouzi et al., 2017; Thevs et al., 2012; Zhao et al., 2024). Hairy root systems *in vitro* allow flavonoid production within 30 days (Zhang et al., 2022a). Variability among genotypes affects fiber and leaf yield, suggesting opportunities for further optimization (Zhao et al., 2024). With optimized cultivation in place, exploring genomic diversity and functional traits can accelerate breeding for improved stress resilience and productivity.

## 5 Challenges and future perspectives

This section outlines practical next steps to move *Apocynum* from research into sustained use. We pair technical gaps with socio-economic and policy actions that enable scale, including clear market entry paths, community adoption models, and fixes for institutional bottlenecks that slow implementation.

### 5.1 Challenges

Despite growing recognition of *Apocynum* for arid ecosystems, several barriers limit scale-up. Agronomically, there are no optimized management packages for diverse dryland environments, and multi-location trials beyond China are scarce. Mechanization is limited. Genetically, within- and across-species variation is poorly characterized, which restricts breeding for fiber yield, flavonoid content, and phytoremediation capacity. Propagation and cultivation protocols are not standardized or cost-effective across site types, constraining scalability. Evidence on stress tolerance remains fragmented across studies, with weak links from physiology and molecular markers to field performance. Gene-editing tools such as CRISPR require species-specific protocols, biosafety assessment, and regulatory pathways before routine use.

#### 5.1.1 Genetic tools and model-organism status of *Apocynum venetum*

In established models such as *Arabidopsis thaliana* and *Oryza sativa*, combined and multifactorial abiotic stresses (for example drought × salinity, heat × salinity) have been dissected using large mutant resources, mapping populations, and time resolved omics, yielding detailed regulatory maps for multi stress acclimation (Jiang et al., 2025c; Saini et al., 2025; Secomandi et al., 2025; Yaschenko et al., 2025; Zandalinas and Mittler, 2022). These insights largely come from glycophytes with limited intrinsic tolerance to extreme arid–saline habitats, which constrains direct translation to restoration contexts. By contrast, Av and Ap combine naturally high drought–salinity tolerance with emerging genomic and physiological resources, but currently lack the mature genetic infrastructure that defines classical model organisms (Abubakar et al., 2022; Thevs et al., 2012; Xiang et al., 2023). For *Apocynum*, the strongest assets are genome and stress response profiling. Chloroplast genomes, chromosome scale nuclear assemblies and comparative genome–metabolome analyses now resolve major phenylpropanoid and flavonoid pathways, ion homeostasis modules and other stress related networks in A*v* and *Ap* (Dorjee et al., 2024; Gao et al., 2023a, 2019; Guo et al., 2022; Xiang et al., 2023; Xie et al., 2024a; Zheng et al., 2022). Transcriptomic, proteomic and metabolomic studies under salt, osmotic, and combined constraints highlight NAC, WRKY and MYB transcription factors, ion transporters such as ApHKT1 and NHX type antiporters, and flavonoid biosynthetic genes including *AvF3H* and *AvFLS* as candidates for drought–salinity tolerance (Chen et al., 2020; Huang et al., 2023; Li et al., 2023c; Wu et al., 2022; Xu et al., 2021; Zhang et al., 2023; Zhao et al., 2025; Zhen et al., 2024). A concise comparison of genetic and functional resources for *Apocynum*, *Arabidopsis* and rice is provided in **Supplementary Table S1**. This table juxtaposes reference genome assemblies, stress-related transcriptomic and proteomic resources, functional gene validation tools, stress-gene databases, high-throughput phenotyping platforms, marker-assisted mapping, gene-editing and transformation systems, and mutant libraries for *Av*/*Ap*, *Arabidopsis thaliana*, and *Oryza sativa*, clarifying both the emerging strengths of *Apocynum* and the remaining gaps relative to classical model species.

The key gap, relative to Arabidopsis and rice, is in genetic tool development, mutant resources and transformation systems. No public large-scale mutant libraries (T DNA, transposon, chemical, or fast neutron) or routine high-efficiency stable transformation and CRISPR-based genome editing protocols are yet available for *Av* or *Ap* (Abubakar et al., 2022; Thevs et al., 2012; Xiang et al., 2023). Consequently, candidate genes from omics studies are usually tested via expression profiling or heterologous overexpression rather than loss or gain of function analysis in *Apocynum*, in sharp contrast to *Arabidopsis* and rice, where forward and reverse genetics and genome editing are standard in combined stress research (Jiang et al., 2025c; Saini et al., 2025; Yaschenko et al., 2025). Thus, *Av* is currently best viewed as a stress resilient, omics enabled halophyte with emerging model features rather than a full genetic model. From a practical standpoint, future work should prioritize (i) robust Agrobacterium-mediated stable transformation in at least one Av and one Ap genotype, (ii) pilot mutagenized or insertional populations with basic sequence indexing, and (iii) CRISPR based editing of a small set of well-characterized candidate genes, integrated with existing Arabidopsis and rice frameworks. Together, these steps would directly address the current tool gaps and move *Apocynum* toward a genetically tractable model for combined stress research.

### 5.2 Genomics

Priorities include chromosome-level assemblies for key species, tissue- and stage-specific transcriptomes under single and combined stresses, and network inference that connects transcription factors to ion transporters and flavonoid enzymes. Community benchmarks should report effect sizes and fold-changes under clearly defined salinity and drought regimes, with shared metadata and code for reproducibility. Comparative genomics across *Av*, *Ap*, and other *Apocynum* species can identify conserved and lineage-specific stress modules to guide marker development.

### 5.3 Agronomy

Field protocols should test sowing windows, saline irrigation thresholds, and deficit-irrigation schedules across soils, with standard reporting of K^+^ to Na^+^ ratios, water-use efficiency, survival, and yield. Validate low-cost establishment at scale, including intercropping on dunes, living windbreaks, and residue management. Evaluate biochar, Plant growth-promoting rhizobacteria (PGPR), and nanoparticle seed treatments side-by-side in common trials that track fiber quality and bioactive profiles, while monitoring safety and environmental compatibility.

### 5.4 Bioeconomy

Develop full-plant biorefinery pathways: stems to technical fiber or nanocellulose, leaves to standardized flavonoid extracts, residues to energy or biochar. Report cradle-to-gate metrics for carbon storage, water use, and unit costs to benchmark *Apocynum* against existing crops. Build supply-chain pilots with quality standards for fiber and phytochemicals and explore under-studied metabolites such as isoquercitrin for pharmaceutical and nutraceutical markets. Market entry can begin with a focused bundle that includes technical fiber specifications for textiles and composites, standardized leaf-extract profiles for flavonoids, and verified biomass for restoration or remediation contracts. Early pilots can reduce risk by using offtake agreements with local processors, small purchase guarantees for first harvests, and quality-testing hubs that issue lot certificates and price to quality.

### 5.5 Policy

Focus on varietal registration and certified seed systems, extension packages for saline and arid zones, and incentives that reward restoration outcomes, together with product value. Align land-use rules to favor planting on marginal lands without competing with food crops, and support regional processing hubs to reduce transport costs. Pilot data-driven programs that pair ecological indicators with household income metrics to test adoption at the community scale. Implementation often stalls due to fragmented permits, unclear land tenure, and limited saline-agriculture services. A practical path is community-based adoption that registers genotype-specific seed, trains local nurseries for cutting and rhizome supply, and pairs producer groups with extension agents delivering one management package per soil class. Provincial programs can lower barriers with small matching grants for drip kits, shared retting or drying facilities, and outcome payments tied to verified fiber quality, leaf-flavonoid standards, or land-stabilization indicators.

## 6 Conclusion

*Apocynum* species, notably *Av* and *Ap*, represent stress-adapted halophytes uniquely adapted to withstand combined drought and salinity stress through coordinated morphological, physiological, biochemical, and molecular mechanisms. Their robust multiscale stress responses, including osmotic adjustment, precise regulation of ion homeostasis, enhanced antioxidant activity, and dynamic gene expression mediated by key transcription factors and phytohormones, underline their potential as model systems for studying abiotic stress resilience in non-model species. Beyond their intrinsic biological resilience, *Apocynum* species deliver substantial ecological benefits essential for restoring degraded drylands. These include improving soil structure, promoting microbial biodiversity, reducing erosion, enhancing carbon sequestration, and facilitating phytoremediation. Economically, they offer significant bioeconomic opportunities through fiber production, pharmaceutical applications, and other bioactive compound utilizations, directly contributing to the sustainable development of arid and semi-arid regions. Integrating *Apocynum* cultivation into climate-smart agricultural practices and circular bioeconomy frameworks represents a pragmatic strategy for simultaneously addressing environmental restoration and socio-economic sustainability. Future research efforts should prioritize advanced molecular tools and omics-based approaches to unravel comprehensive stress adaptation mechanisms, optimize scalable cultivation techniques, and evaluate ecological impacts rigorously. Further development and deployment of *Apocynum* as a climate-smart crop can significantly contribute to global sustainability goals, particularly Sustainable Development Goals (SDGs) 13 (Climate Action) and 15 (Life on Land). Thus, leveraging *Apocynum*’s intrinsic adaptations and integrating interdisciplinary research can establish a resilient framework for sustainable agricultural practices and ecosystem restoration under the growing challenge of combined abiotic stress. While the current evidence on *Apocynum* is primarily centered in China, validating its ecological and economic potential across diverse arid and semi-arid regions is a crucial next step. Establishing global partnerships, running multi-location field trials, and integrating region-specific agronomic data will be essential to adapt *Apocynum*-based systems to varied environmental, socio-economic, and policy contexts. These efforts will support broader scalability and real-world impact in addressing climate resilience and land degradation.
