# Supplementary material for: Multiscale responses to combined drought and salinity stress in Apocynum: a model for climate-smart dryland restoration
Source: Front Plant Sci. 2026 Jan 9;16:1664532. doi: 10.3389/fpls.2025.1664532 (PMC12827735; doi:10.3389/fpls.2025.1664532)
Supplement: Supplementary file 1 [file Table1.docx]

Supplementary Material

# Supplementary Tables

**Supplementary Table 1.** Comparative genomics and functional resources for *Apocynum*, *Arabidopsis thaliana,* and *Oryza sativa* relevant to combined drought and salinity stress research.

| **Tool / Resource** | ***Apocynum* (*Av* /*Ap*)** | ***Arabidopsis thaliana*** | ***Oryza sativa*** | **References** |
| --- | --- | --- | --- | --- |
| Reference genome assemblies | Chromosome-scale genomes for *Av* and *Ap*; enable phylogeny, bast fibre, and flavonoid/stress-gene analyses. | Complete reference genome (TAIR) with high-quality annotation and extensive resequencing panels. | Nipponbare IRGSP-1.0 reference plus large resequencing panels (e.g. 3K rice genomes) and multiple cultivar assemblies. | (3,000 Rice Genomes Project, 2014; Dorjee et al., 2024; Kawahara et al., 2013; Ohyanagi et al., 2006; Rice Annotation Project; The Arabidopsis Genome Initiative, 2000; Xie et al., 2024) |
| Transcriptomics (RNA-seq, microarray) | Multiple RNA-seq datasets for salt and drought stress; de novo transcriptomes and WGCNA networks identify salt-tolerance candidates. | Large-scale microarray and RNA-seq for drought, cold, and salinity; comprehensive abiotic-stress gene expression atlases (e.g. AtSRGA). | Numerous RNA-seq and meta-analyses for drought, salinity, and combined stresses; rice-specific stress expression databases and meta-resources. | (Azad et al., 2024; Fukuda et al., 2025; Sandhu et al., 2017; Seki et al., 2002; Xu et al., 2021; Yuan et al., 2020; Zhen et al., 2024) |
| Proteomics / metabolomics | Metabolomics and iTRAQ proteomics under salt stress; element–metabolite networks under combined salt–drought. | Numerous abiotic-stress metabolomics and phenomics studies; stress responses often analysed in multi-omics frameworks. | Integrated transcriptomic, metabolomic and pathway-level resources summarised for multiple abiotic stresses. | (Chen et al., 2019; Chen et al., 2020; Iqbal et al., 2021; Jiang et al., 2025; Liu et al. 2016; Rahman et al., 2022) |
| Functional gene validation (mutants, overexpression, qPCR) | Mostly expression-based candidate validation (qPCR, correlation with stress tolerance); stable transgenics still rare or not reported. | Extensive mutant and overexpression work for abiotic-stress regulators (e.g. SOS1 Na⁺/H⁺ antiporter, many TFs) and combined-stress GWAS/QTL follow-ups. | Numerous QTL/gene validations for drought and salinity tolerance, including functional tests in transgenic or gene-edited lines. | (Alam et al., 2022; Awlia et al., 2021; Shi et al., 2000; Zhang et al., 2019) |
| Stress-responsive gene databases / tools | None species-specific reported so far. | Arabidopsis stress-gene databases and atlases (AtSR, AtSRGA) with curated stress-responsive genes and co-expression information. | Rice stress-gene resources such as STIFDB2, RiceSRTFDB, APRegNet and metabolic network databases (OryzaCyc). | (Borkotoky et al., 2013; Fukuda et al., 2025; Naika et al., 2013; Shaik and Ramakrishna, 2013) |
| High-throughput phenotyping | Not yet reported for Apocynum (at least in the peer-reviewed literature). | Automated imaging platforms (e.g. Phenoscope) and phenotyping pipelines for salinity and drought responses, used with GWAS/QTL. | Greenhouse and field-based high-throughput imaging for salinity and drought; platforms used to map salinity loci and yield components, mostly for single stresses. | (Al-Tamimi et al., 2016; Awlia et al., 2016; Hairmansis et al., 2014; Tisné et al., 2013) |
| Marker-assisted selection / genetic mapping (GWAS, QTL) | No GWAS/QTL mapping reported yet for Apocynum stress tolerance. | GWAS/QTL for abiotic and combined stress responses, including distinct architectures for single vs combined stresses. | Extensive GWAS/QTL for drought and salinity; polymorphism scans and QTL-based candidate gene mining for marker-assisted breeding. | (Abhayawickrama et al., 2020; Al-Tamimi et al., 2016; Awlia et al., 2021; Dávila Olivas et al., 2017; Jian et al. 2018; Kumar et al., 2015; Kumar et al., 2018) |
| Gene editing (CRISPR) | No *Apocynum* CRISPR system reported so far. | CRISPR/Cas9 widely used to dissect abiotic-stress regulators (e.g. HSFA6a/b, WRKY3/4) and ABA/stress pathways. | CRISPR/Cas9 and related systems used for single- and multiplex editing of stress-tolerance and yield loci (e.g. OsRR22, OsbHLH024, various PUB and stress-related genes). | (Alam et al., 2022; Li et al., 2021; Li et al., 2022; Wang et al., 2020; Zafar et al., 2020; Zhang et al., 2019) |
| Stable transformation | Only sporadic reports; no routine high-efficiency stable transformation system yet. | Simple, routine Agrobacterium-mediated stable transformation (floral-dip method) widely used for nuclear transgenics. | Mature Agrobacterium-mediated transformation for japonica and many indica cultivars using immature embryos or callus. | (Ali et al. 2022; Chen et al. 2023; Haque et al. 2025; Liu et al. 2025) |
| Mutant libraries (EMS, T-DNA, transposon, FN, CRISPR collections) | No public large-scale mutant collection reported. | Massive T-DNA and transposon resources (SALK, GABI-Kat, RIKEN Ds, etc.) covering most genes; detailed user guides and quality control analyses. | Large T-DNA insertion populations and fast-neutron mutant panels for rice cultivars; activation-tagging and sequence-indexed mutant sets. | (Fu et al., 2009; Jeong et al., 2002; Li et al., 2018; O’Malley & Ecker, 2010; O’Malley et al. 2015; Wolella et al. 2025; Zhang et al. 2006) |
| Hairy root / transient assays (rapid functional tests) | No published Apocynum hairy-root or protoplast system yet. | Robust transient expression via mesophyll protoplasts; hairy-root protocols available for targeted root studies. | Efficient rice protoplast isolation and transient expression systems for rapid gene function and promoter assays. | (He et al., 2016; Page et al., 2019; Paul et al., 2022; Poddar et al., 2020) |

**References**

3,000 Rice Genomes Project. (2014). The 3,000 rice genomes project. Gigascience, 3(1), 2047-217X. doi: <https://doi.org/10.1186/2047-217X-3-7>

Abhayawickrama, B., Gimhani, D., Kottearachchi, N., Herath, V., Liyanage, D., & Senadheera, P. (2020). In Silico Identification of QTL-Based Polymorphic Genes as Salt-Responsive Potential Candidates through Mapping with Two Reference Genomes in Rice. Plants, 9(2), 233. doi: <https://doi.org/10.3390/plants9020233>

Al-Tamimi, N., Brien, C., Oakey, H., Berger, B., Saade, S., Ho, Y. S., ... & Negrão, S. (2016). Salinity tolerance loci revealed in rice using high-throughput non-invasive phenotyping. Nature communications, 7(1), 13342. doi: <https://doi.org/10.1038/ncomms13342>

Alam, M. S., Kong, J., Tao, R., Ahmed, T., Alamin, M., Alotaibi, S. S., Abdelsalam, N. R., and Xu, J.-H. (2022). CRISPR/Cas9 mediated knockout of the OsbHLH024 transcription factor improves salt stress resistance in rice (Oryza sativa L.). Plants 11, 1184. Doi: <https://doi.org/10.3390/plants11091184>

Ali, I., Sher, H., Ali, A., Hussain, S., & Ullah, Z. (2022). Simplified floral dip transformation method of Arabidopsis thaliana.. Journal of microbiological methods, 106492. doi:<https://doi.org/10.1016/j.mimet.2022.106492>.

Awlia, M., Nigro, A., Fajkus, J., Schmoeckel, S. M., Negrão, S., Santelia, D., Trtílek, M., Tester, M., Julkowska, M. M., & Panzarová, K. (2016). High-throughput non-destructive phenotyping of traits that contribute to salinity tolerance in Arabidopsis thaliana. Frontiers in Plant Science, 7, 1414. doi:’ <https://doi.org/10.3389/fpls.2016.01414>

Awlia, M., Alshareef, N., Saber, N., Korte, A., Oakey, H., Panzarová, K., Trtílek, M., Negrão, S., Tester, M., and Julkowska, M. M. (2021). Genetic mapping of the early responses to salt stress in Arabidopsis thaliana. The Plant Journal 107(2), 544–563. doi: <https://doi.org/10.1111/tpj.15310>

Azad, M., Tohidfar, M., Ghanbari Moheb Seraj, R., Mehralian, M., & Esmaeilzadeh-Salestani, K. (2024). Identification of responsive genes to multiple abiotic stresses in rice (Oryza sativa): a meta-analysis of transcriptomics data. Scientific reports, 14(1), 5463. doi: <https://doi.org/10.1038/s41598-024-54623-7>

Borkotoky, S., Saravanan, V., Jaiswal, A., Das, B., Selvaraj, S., Murali, A., & Lakshmi, P. T. V. (2013). The Arabidopsis stress responsive gene database. International journal of plant genomics, 2013(1), 949564. doi: <https://doi.org/10.1155/2013/949564>

Chen, C., Liu, H., Wang, C., Liu, Z., Liu, X., Zou, L., Zhao, H., Yan, Y., Shi, J., and Chen, S. (2019). Metabolomics characterizes metabolic changes of Apocyni veneti folium in response to salt stress. Plant Physiology and Biochemistry 144, 187–196. doi: <https://doi.org/10.1016/j.plaphy.2019.09.043>

Chen, C., Liu, H., Wang, C., Liu, Z., Liu, X., Zou, L., ... & Chen, S. (2019). Metabolomics characterizes metabolic changes of Apocyni Veneti Folium in response to salt stress. Plant Physiology and Biochemistry, 144, 187-196. doi: <https://doi.org/10.1016/j.plaphy.2019.09.043>

Chen, C., Wang, C., Liu, Z., Cai, Z., Hua, Y., Mei, Y., ... & Liu, X. (2020). iTRAQ-based proteomic technique provides insights into salt stress responsive proteins in Apocyni Veneti Folium (Apocynum venetum L.). Environmental and Experimental Botany, 180, 104247. doi: <https://doi.org/10.1016/j.envexpbot.2020.104247>

Chen, K., Ye, C., Guo, J., Chen, D., Guo, T., Liu, J., Liu, C., & Zhou, X. (2023). Agrobacterium-mediated transformation efficiency and grain phenotypes in six indica and japonica rice cultivars. Seed Biology. doi: <https://doi.org/10.48130/seedbio-2023-0004>

Davila Olivas, N. H., Kruijer, W., Gort, G., Wijnen, C. L., van Loon, J. J., & Dicke, M. (2017). Genome‐wide association analysis reveals distinct genetic architectures for single and combined stress responses in Arabidopsis thaliana. New Phytologist, 213(2), 838-851. doi: <https://doi.org/10.1111/nph.14165>

Dorjee, T., Tan, J., Zuo, Q., Zheng, L., Liu, Q., Sun, H., Zhou, Y., & Gao, F. (2024). Chromosome-scale genome analysis of Apocynum venetum sheds light on Apocynum phylogenetics, bast fibre development, and flavonoid synthesis. Industrial Crops and Products, 212, 118325. doi: <https://doi.org/10.1016/j.indcrop.2024.118325>

Fukuda, Y., Kawaguchi, K., & Fukushima, A. (2025). AtSRGA: A shiny application for retrieving and visualizing stress-responsive genes in Arabidopsis thaliana. Plant Physiology, 197(4), kiaf105. doi: <https://doi.org/10.1093/plphys/kiaf105>

Fu, J., Keurentjes, J. J. B., Bouwmeester, H., America, T., Verstappen, F. W. A., Ward, J. L., Beale, M. H., de Vos, R. C. H., Dijkstra, M., Scheltema, R. A., Johannes, F., Koornneef, M., Vreugdenhil, D., Breitling, R., & Jansen, R. C. (2009). System-wide molecular evidence for phenotypic buffering in Arabidopsis. Nature Genetics, 41(2), 166–167. doi: <https://doi.org/10.1038/ng.308>

Hairmansis, A., Berger, B., Tester, M., & Roy, S. J. (2014). Image-based phenotyping for non-destructive screening of different salinity tolerance traits in rice. Rice, 7(1), 16. doi: <https://doi.org/10.1186/s12284-014-0016-3>

Haque, M., Okumura, Y., & Kidou, S. (2025). Highly efficient direct seed transformation protocol for japonica rice (Oryza sativa L.) by Agrobacterium tumefaciens: overcoming the complexity of callus regeneration and avoiding the occurrence of somaclonal-mutations.. Transgenic research, 34 1, 36. doi: <https://doi.org/10.1007/s11248-025-00453-w>.

He, F., Chen, S., Ning, Y., & Wang, G. L. (2016). Rice (Oryza sativa) protoplast isolation and its application for transient expression analysis. Current protocols in plant biology, 1(2), 373-383. doi: <https://doi.org/10.1002/cppb.20026>

Iqbal, Z., Iqbal, M. S., Khan, M. I. R., & Ansari, M. I. (2021). Toward integrated multi-omics intervention: rice trait improvement and stress management. Frontiers in Plant Science, 12, 741419. doi: <https://doi.org/10.3389/fpls.2021.741419>

Jeong, D.-H., An, S., Kang, H.-G., Moon, S., Han, J.-J., Park, S., Lee, H. S., An, K., & An, G. (2002). T-DNA insertional mutagenesis for activation tagging in rice. Plant Physiology, 130(4), 1636–1644. doi: <https://doi.org/10.1104/pp.014357>

Jian, L. I. U., Jun, Z. H. A. O., Liang-fang, D. A. I., Ya-ling, C. H. E. N., Biao-lin, H. U., & Jian-kun, X. I. E. (2018). Rapid mapping of candidate genes for cold tolerance in Oryza rufipogon Griff. by QTL-seq of seedlings. Journal of integrative agriculture, 17(2), 265-275. doi: <https://doi.org/10.1016/S2095-3119(17)61712-X>

Jiang, L., Zhao, Y., Jiang, Z., Wu, Y., Wen, B., Zhang, S., Zhan, J., and Su, N. (2025). Comprehensive analysis of element and metabolite content between the seeds of Apocynum venetum and Apocynum pictum provides new sights for the salt tolerance in Apocynum. Frontiers in Plant Science 16, 1611975. doi: <https://doi.org/10.3389/fpls.2025.1611975>

Kawahara, Y., de la Bastide, M., Hamilton, J. P., Kanamori, H., McCombie, W. R., Ouyang, S., Schwartz, D. C., Tanaka, T., Wu, J., Zhou, S., Childs, K. L., Davidson, R. M., Lin, H., Quesada-Ocampo, L., Vaillancourt, B., Sakai, H., Lee, S. S., Kim, J., Numa, H., Itoh, T., Buell, C. R., and Matsumoto, T. (2013). Improvement of the Oryza sativa Nipponbare reference genome using next generation sequence and optical map data. Rice 6, 4. doi: <https://doi.org/10.1186/1939-8433-6-4>

Kumar, A., Sandhu, N., Dixit, S., Yadav, S., Swamy, B. P. M., & Shamsudin, N. A. A. (2018). Marker-assisted selection strategy to pyramid two or more QTLs for quantitative trait-grain yield under drought. Rice, 11(1), 35. doi: <https://doi.org/10.1186/s12284-018-0227-0>

Kumar, V., Singh, A., Mithra, S. V. A., Krishnamurthy, S. L., Parida, S. K., Jain, S., Tiwari, K. K., Kumar, P., Rao, A. R., Sharma, S. K., Khurana, J. P., Singh, N. K., & Mohapatra, T. (2015). Genome-wide association mapping of salinity tolerance in rice (Oryza sativa). DNA Research, 22(2), 133–145. doi: <https://doi.org/10.1093/dnares/dsu046>

Li, B., Du, X., Fei, Y., Wang, F., Xu, Y., Li, X., ... & Yang, J. (2021). Efficient breeding of early-maturing rice cultivar by editing PHYC via CRISPR/Cas9. Rice, 14(1), 86. doi: <https://doi.org/10.1186/s12284-021-00527-3>

Li, Y., Li, X., Fu, D., & Wu, C. (2018). Panicle Morphology Mutant 1 (PMM1) determines the inflorescence architecture of rice by controlling brassinosteroid biosynthesis. BMC Plant Biology, 18(1), 348. doi: <https://doi.org/10.1186/s12870-018-1577-x>

Li, Y., Wu, X, Zhang, Y., & Zhang, Q. (2022). CRISPR/Cas genome editing improves abiotic and biotic stress tolerance of crops. Frontiers in Genome Editing, 4, 987817. doi: <https://doi.org/10.3389/fgeed.2022.987817>

Liu, M., Huang, T., Wang, Y., Wang, S., Wu, C., Kuo, C., & Lai, E. (2025). Floral Stage Optimization and Immune Evasion Enhance Agrobacterium-Mediated Genome Editing in Arabidopsis. bioRxiv. doi: <https://doi.org/10.1101/2025.05.07.652770>.

Liu, X. X., Tang, L., Ge, R., Li, J. K., Kang, Y., Zhu, M. X., ... & Hao, X. L. (2016). iTRAQ-based quantitative proteomic analysis of the anti-apoptotic effect of hyperin, which is mediated by Mcl-1 and Bid, in H2O2-injured EA. hy926 cells. International Journal of Molecular Medicine, 37(4), 1083-1090. doi: <https://doi.org/10.3892/ijmm.2016.2510>

Naika, M., Shameer, K., Mathew, O. K., Gowda, R., & Sowdhamini, R. (2013). STIFDB2: an updated version of plant stress-responsive transcription factor database with additional stress signals, stress-responsive transcription factor binding sites and stress-responsive genes in Arabidopsis and rice. Plant and Cell Physiology, 54(2), e8-e8. doi: <https://doi.org/10.1093/pcp/pcs185>

O’Malley, R. C., & Ecker, J. R. (2010). Linking genotype to phenotype using the Arabidopsis unimutant collection. The Plant Journal, 61(6), 928-940. doi: <https://doi.org/10.1111/j.1365-313X.2010.04119.x>

O’Malley, R. C., Barragan, C. C., & Ecker, J. R. (2015). A user’s guide to the Arabidopsis T-DNA insertion mutant collections. In Plant functional genomics: methods and protocols (pp. 323-342). New York, NY: Springer New York. doi: <https://doi.org/10.1007/978-1-4939-2444-8_16>

Ohyanagi, H., Tanaka, T., Sakai, H., Shigemoto, Y., Yamaguchi, K., Habara, T., Fujii, Y., Antonio, B. A., Nagamura, Y., Imanishi, T., Ikeo, K., Itoh, T., Gojobori, T., and Sasaki, T. (2006). The Rice Annotation Project Database (RAP-DB): hub for Oryza sativa ssp. japonica genome information. Nucleic Acids Res. 34, D741–D744. doi: <https://doi.org/10.1093/nar/gkj094>

Page, M. T., Parry, M. A., & Carmo‐Silva, E. (2019). A high‐throughput transient expression system for rice (Vol. 42, No. 7, pp. 2057-2064). doi: <https://doi.org/10.1111/pce.13542>

Paul, P., Majumdar, S., & Jha, S. (2022). A simple and efficient protocol for hairy root culture of Arabidopsis thaliana. Plant Cell, Tissue and Organ Culture (PCTOC), 150(1), 105-112. doi: <https://doi.org/10.1007/s11240-022-02248-x>

Poddar, S., Tanaka, J., Cate, J. H., Staskawicz, B., & Cho, M. J. (2020). Efficient isolation of protoplasts from rice calli with pause points and its application in transient gene expression and genome editing assays. Plant Methods, 16(1), 151. doi: <https://doi.org/10.1186/s13007-020-00692-4>

Rahman, M. U., Zulfiqar, S., Raza, M. A., Ahmad, N., & Zhang, B. (2022). Engineering abiotic stress tolerance in crop plants through CRISPR genome editing. Cells, 11(22), 3590. doi: <https://doi.org/10.3390/cells11223590>

Rice Annotation Project. (2007). The rice annotation project database (RAP-DB): 2008 update. Nucleic Acids Research, 36(suppl_1), D1028-D1033. doi: <https://doi.org/10.1093/nar/gkm978>

Rosso, M. G., Li, Y., Strizhov, N., Reiss, B., Dekker, K., & Weisshaar, B. (2003). An Arabidopsis thaliana T-DNA mutagenized population (GABI-Kat) for flanking sequence tag-based reverse genetics. Plant molecular biology, 53(1), 247-259. doi: <https://doi.org/10.1023/B:PLAN.0000009297.37235.4a>

Sandhu, M., Sureshkumar, V., Prakash, C., Dixit, R., Solanke, A. U., Sharma, T. R., Mohapatra, T., and Mithra, A. M. S. V. (2017). RiceMetaSys for salt and drought stress responsive genes in rice: a web interface for crop improvement. BMC Bioinformatics 18, 432. doi: <https://doi.org/10.1186/s12859-017-1846-y>

Seki, M., Narusaka, M., Ishida, J., Nanjo, T., Fujita, M., Oono, Y., Kamiya, A., Nakajima, M., Enju, A., Sakurai, T., Satou, M., Akiyama, K., Taji, T., Yamaguchi-Shinozaki, K., Carninci, P., Kawai, J., Hayashizaki, Y., and Shinozaki, K. (2002). Monitoring the expression profiles of 7000 Arabidopsis genes under drought, cold and high-salinity stresses using a full-length cDNA microarray. The Plant Journal 31(3), 279–292. doi: <https://doi.org/10.1046/j.1365-313X.2002.01359.x>

Shaik, R., & Ramakrishna, W. (2013). Genes and co-expression modules common to drought and bacterial stress responses in Arabidopsis and rice. PloS one, 8(10), e77261. doi: <https://doi.org/10.1371/journal.pone.0077261>

Shi, H., Ishitani, M., Kim, C., & Zhu, J. K. (2000). The Arabidopsis thaliana salt tolerance gene SOS1 encodes a putative Na+/H+ antiporter. Proceedings of the national academy of sciences, 97(12), 6896-6901. doi: <https://doi.org/10.1073/pnas.120170197>

The Arabidopsis Genome Initiative. (2000). Analysis of the genome sequence of the flowering plant Arabidopsis thaliana. Nature, 408, 796–815. doi: <https://doi.org/10.1038/35048692>

Thoen, M. P. M., Davila Olivas, N. H., Kloth, K. J., Coolen, S., Huang, P.-P., Aarts, M. G. M., Bac-Molenaar, J. A., Bakker, J., Bouwmeester, H. J., Broekgaarden, C., Bucher, J., Busscher-Lange, J., Cheng, X., Fradin, E. F., Jongsma, M. A., Julkowska, M. M., Keurentjes, J. J. B., Ligterink, W., Pieterse, C. M. J., Ruyter-Spira, C., Smant, G., Testerink, C., Usadel, B., van Loon, J. J. A., van Pelt, J. A., van Schaik, C. C., van Wees, S. C. M., Visser, R. G. F., Voorrips, R., Vosman, B., Vreugdenhil, D., Warmerdam, S., Wiegers, G. L., van Heerwaarden, J., Kruijer, W., van Eeuwijk, F. A., & Dicke, M. (2017). Genetic architecture of plant stress resistance: multi-trait genome-wide association mapping. New Phytologist, 213(3), 1346-1362. doi: <https://doi.org/10.1111/nph.14220>

Tisné, S., Serrand, Y., Bach, L., Gilbault, E., Ben Ameur, R., Balasse, H., Voisin, R., Bouchez, D., Durand-Tardif, M., Guerche, P., Chareyron, G., Da Rugna, J., Camilleri, C., & Loudet, O. (2013). Phenoscope: An automated large-scale phenotyping platform offering high spatial homogeneity. The Plant Journal, 74(3), 534-544. doi: <https://doi.org/10.1111/tpj.12131>

Wang, S., Yang, Y., Guo, M., Zhong, C., Yan, C., & Sun, S. (2020). Targeted mutagenesis of amino acid transporter genes for rice quality improvement using the CRISPR/Cas9 system. The Crop Journal, 8(3), 457-464. doi: <https://doi.org/10.1016/j.cj.2020.02.005>

Wolella, E. K., Cheng, Z., Li, M., Xia, D., Zhang, J., Duan, L., Liu, L., Li, Z., & Zhang, J. (2025). Large-Scale Rice Mutant Establishment and High-Throughput Mutant Manipulation Help Advance Rice Functional Genomics. Plants, 14(10), 1492. doi: <https://doi.org/10.3390/plants14101492>

Xie, W., Bai, B., & Wang, Y. (2024). Chromosome-scale genome assembly of Apocynum pictum, a drought-tolerant medicinal plant from the Tarim Basin. G3: Genes, Genomes, Genetics, 14(12), jkae237. doi: <https://doi.org/10.1093/g3journal/jkae237>

Xu, Z., Wang, M., Ren, T., Li, K., Li, Y., Marowa, P., & Zhang, C. (2021). Comparative transcriptome analysis reveals the molecular mechanism of salt tolerance in Apocynum venetum. Plant Physiology and Biochemistry, 167, 816-830. doi: <https://doi.org/10.1016/j.plaphy.2021.08.043>

Yuan, N., Li, M., & Jia, C. (2020). De novo transcriptome assembly and population genetic analyses of an important coastal shrub, Apocynum venetum L. BMC Plant Biology, 20(1), 408. doi: <https://doi.org/10.1186/s12870-020-02626-7>

Zafar, S. A., Zaidi, S. S. E. A., Gaba, Y., Singla-Pareek, S. L., Dhankher, O. P., Li, X., Mansoor, S., and Pareek, A. (2020). Engineering abiotic stress tolerance via CRISPR/Cas-mediated genome editing. Journal of Experimental Botany, 71(2), 470–479. doi: <https://doi.org/10.1093/jxb/erz476>

Zhang, A., Liu, Y., Wang, F., Li, T., Chen, Z., Kong, D., Bi, J., Zhang, F., Luo, X., Wang, J., Tang, J., Yu, X., Liu, G., and Luo, L. (2019). Enhanced rice salinity tolerance via CRISPR/Cas9-targeted mutagenesis of the OsRR22 gene. Molecular Breeding 39(3), 47. doi: <https://doi.org/10.1007/s11032-019-0954-y>

Zhang, J., Li, C., Wu, C., Xiong, L., Chen, G., Zhang, Q., & Wang, S. (2006). RMD: a rice mutant database for functional analysis of the rice genome. Nucleic acids research, 34(suppl_1), D745-D748. doi: <https://doi.org/10.1093/nar/gkj016>

Zhang, L., Hu, B., Deng, K., Gao, X., Sun, G., Zhang, Z., ... & Chu, C. (2019). NRT1. 1B improves selenium concentrations in rice grains by facilitating selenomethinone translocation. Plant biotechnology journal, 17(6), 1058-1068. doi: <https://doi.org/10.1111/pbi.13037>

Zhen, X., Liu, X., Zhang, X., Luo, S., Wang, W., & Wan, T. (2024). Identification of core genes involved in the response of Apocynum venetum to salt stress based on transcriptome sequencing and WGCNA. Plos one, 19(4), e0300277. doi: <https://doi.org/10.1371/journal.pone.0300277>
